# Effectiveness of a brief behavioural intervention on psychological distress among women with a history of gender-based violence in urban Kenya: A randomised clinical trial

**DOI:** 10.1371/journal.pmed.1002371

**Published:** 2017-08-15

**Authors:** Richard A. Bryant, Alison Schafer, Katie S. Dawson, Dorothy Anjuri, Caroline Mulili, Lincoln Ndogoni, Phiona Koyiet, Marit Sijbrandij, Jeannette Ulate, Melissa Harper Shehadeh, Dusan Hadzi-Pavlovic, Mark van Ommeren

**Affiliations:** 1 School of Psychology, University of New South Wales, Sydney, New South Wales, Australia; 2 Westmead Institute for Medical Research, Sydney, New South Wales, Australia; 3 World Vision International, Monrovia, California, United States of America; 4 World Vision Kenya, Nairobi, Kenya; 5 Psychosocial Support Centre, Nairobi, Kenya; 6 Vrije Universiteit, Amsterdam, Netherlands; 7 World Vision Canada, Missossauga, Ontario, Canada; 8 Department of Mental Health and Substance Abuse, World Health Organization, Geneva, Switzerland; Massachusetts General Hospital, UNITED STATES

## Abstract

**Background:**

Gender-based violence (GBV) represents a major cause of psychological morbidity worldwide, and particularly in low- and middle-income countries (LMICs). Although there are effective treatments for common mental disorders associated with GBV, they typically require lengthy treatment programs that may limit scaling up in LMICs. The aim of this study was to test the effectiveness of a new 5-session behavioural treatment called Problem Management Plus (PM+) that lay community workers can be taught to deliver.

**Methods and findings:**

In this single-blind, parallel, randomised controlled trial, adult women who had experienced GBV were identified through community screening for psychological distress and impaired functioning in Nairobi, Kenya. Participants were randomly allocated in a 1:1 ratio either to PM+ delivered in the community by lay community health workers provided with 8 days of training or to facility-based enhanced usual care (EUC) provided by community nurses. Participants were aware of treatment allocation, but research assessors were blinded. The primary outcome was psychological distress as measured by the total score on the 12-item General Health Questionnaire (GHQ-12) assessed at 3 months after treatment. Secondary outcomes were impaired functioning (measured by the WHO Disability Adjustment Schedule [WHODAS]), symptoms of posttraumatic stress (measured by the Posttraumatic Stress Disorder Checklist [PCL]), personally identified problems (measured by Psychological Outcome Profiles [PSYCHLOPS]), stressful life events (measured by the Life Events Checklist [LEC]), and health service utilisation. Between 15 April 2015 and 20 August 2015, 1,393 women were screened for eligibility on the basis of psychological distress and impaired functioning. Of these, 518 women (37%) screened positive, of whom 421 (81%) were women who had experienced GBV. Of these 421 women, 209 were assigned to PM+ and 212 to EUC. Follow-up assessments were completed on 16 January 2016. The primary analysis was intention to treat and included 53 women in PM+ (25%) and 49 women in EUC (23%) lost to follow-up. The difference between PM+ and EUC in the change from baseline to 3 months on the GHQ-12 was 3.33 (95% CI 1.86–4.79, *P* = 0.001) in favour of PM+. In terms of secondary outcomes, for WHODAS the difference between PM+ and EUC in the change from baseline to 3-month follow-up was 1.96 (95% CI 0.21–3.71, *P* = 0.03), for PCL it was 3.95 (95% CI 0.06–7.83, *P* = 0.05), and for PSYCHLOPS it was 2.15 (95% CI 0.98–3.32, *P* = 0.001), all in favour of PM+. These estimated differences correspond to moderate effect sizes in favour of PM+ for GHQ-12 score (0.57, 95% CI 0.32–0.83) and PSYCHLOPS (0.67, 95% CI 0.31–1.03), and small effect sizes for WHODAS (0.26, 95% CI 0.02–0.50) and PCL (0.21, 95% CI 0.00–0.41). Twelve adverse events were reported, all of which were suicidal risks detected during screening. No adverse events were attributable to the interventions or the trial. Limitations of the study include no long-term follow-up, reliance on self-report rather than structured interview data, and lack of an attention control condition.

**Conclusions:**

Among a community sample of women in urban Kenya with a history of GBV, a brief, lay-administered behavioural intervention, compared with EUC, resulted in moderate reductions in psychological distress at 3-month follow-up.

**Trial registration:**

Australian New Zealand Clinical Trials Registry ACTRN12614001291673

## Abstract 2

In this clinical trial in Kenya, Richard A. Bryant of the University of New South Wales, Australia, and colleagues show that a brief behavioral intervention of up to five sessions administered by community lay workers can reduce psychological distress among women who have experienced gender-based violence.

## Author summary

**Why was this study done?:**

- Gender-based violence (GBV) is a major public issue that causes a range of mental health problems across the globe.
- Most countries affected by GBV lack the health resources to address these mental health problems because existing treatments for common mental disorders typically require mental health specialists, involve lengthy treatments (which increases costs), and focus only on a single disorder.
- In response to this situation, the World Health Organization has developed a brief psychological intervention that can be delivered by trained lay workers that aims to reduce psychological distress following adversity.
- Our study was designed to test the effectiveness of this intervention, called Problem Management Plus (PM+), in distressed women affected by GBV.

**What did the researchers do and find?:**

- This study screened women in a peri-urban area of Nairobi, Kenya, and subsequently randomised 421 women who displayed distress and impaired functioning to either 5 individual sessions of PM+ or enhanced usual care (EUC).
- Independent assessments indicated that 3 months following treatment, women who received PM+ reported significantly less psychological distress, with a moderate effect relative to EUC.
- Although PM+ also led to greater reductions in posttraumatic stress and functional impairment than EUC, these effects were small.

**What do these findings mean?:**

- This study suggests that lay health workers can be trained in PM+, and they can deliver this intervention in a way that it can improve the mental health of GBV-affected women.
- This brief intervention has the potential to scale up mental health services for GBV-affected women in countries that lack mental health specialists.

## Introduction

One of the most concerning potentially traumatic events worldwide is gender-based violence (GBV), including physical and sexual violence against women by an intimate partner or others [1]. At least one-third of women have experienced GBV [2], which is a global public health issue because of its adverse impacts on physical and mental health [3]. Addressing the mental health needs of women who have experienced GBV is particularly problematic in low- and middle-income countries (LMICs) [3], where mental health services are often unavailable [4]. Social stigma regarding GBV can be so high that affected women are at risk of further violence or abandonment if they disclose the violence [5], and this can impede women’s seeking of assistance [6]. Accordingly, multiple agencies recommend integrating care of women who have experienced GBV into general health services to safely reach a maximum number of affected women [7,8].

Much evidence exists for effective treatment of common mental disorders after trauma, such as depression and posttraumatic stress disorder (PTSD), with most studies using cognitive behaviour therapy (CBT) with a trauma focus [9,10]. One seminal study in the Democratic Republic of the Congo demonstrated that victims of sexual violence can also be treated by non-specialist psychosocial assistants with trauma-focused CBT [10]. A major challenge for the implementation of established psychological interventions among women who have experienced GBV in LMICs, however, is that interventions typically require at least 12 sessions. Lengthy programs can impede implementation in LMICs because they increase the expense of treatment delivery as well as the demands on affected women, who often cannot commit to lengthy programs (for economic and personal reasons). Although there is evidence that advocacy and cognitive behavioural interventions can partially address the occurrence and psychological effects of GBV [11,12] and these need to be scaled up, there is also an urgent need for brief and effective interventions that can be made available without the commitment of more costly or lengthy treatment programs.

To address these challenges, WHO developed a brief psychological intervention, termed Problem Management Plus (PM+), comprising strategies that people without qualifications or experience in mental health can be trained to deliver to reduce common mental disorders following adversity [13,14]. The evidence-based strategies in this program include behavioural activation, problem-solving, accessing social support, and stress reduction [15,16]. These strategies purportedly improve mental health because (a) increasing activity reduces depression [16]; (b) developing the capacity to solve problems is an effective means to improve mental health [15], and can be especially relevant in post-adversity contexts; (c) receiving social support decreases stress responses [17]; and (d) stress reduction techniques reduce anxiety, arguably as a result of arousal reduction [18]. Initial support for this program has come from a trial in primary healthcare clinics in conflict-affected Peshawar, Pakistan, where PM+ resulted in greater reductions in psychological distress and improved functioning than enhanced usual care (EUC), which comprised a 1-day refresher training of primary care physicians on common presentations of anxiety and depression, psychoeducation, supportive counselling, psychotropic medication, and referral [19].

In the context of the need for scalable interventions for women affected by GBV, this study assessed the effectiveness of PM+ to alleviate distress in women who had experienced GBV in peri-urban slums in Nairobi, Kenya, where women are frequently exposed to violence. In this single-blind, parallel, randomised controlled trial, adult women who had experienced GBV and who were impaired by distress received either PM+ delivered by lay community health workers (CHWs) or EUC delivered by qualified community nurses. It was hypothesized that PM+ would reduce psychological distress, impaired functioning, posttraumatic stress, personally identified problems, and health utilisation relative to EUC at the follow-up assessment. The primary outcome, psychological distress, was measured at 3 months rather than immediately posttreatment (7 weeks after baseline assessment) so that the medium-term effects of the PM+ intervention could be determined.

## Methods

### Participants

The trial was approved by the WHO Research Ethics Review Committee (RPC656) and the Great Lakes University Ethics Committee in Kenya. The protocol was registered in the Australian New Zealand Clinical Trials Registry (ACTRN12614001291673) on 10 December 2014. Details of the procedures have been described previously [20]. Participants were recruited by interviewing 1 woman from 1 of every 10 households in peri-urban areas in Nairobi. In the absence of street addresses, assessors were directed to start in the community at a point randomly designated by a supervisor and instructed to visit every 10th house and interview 1 woman. If more than 1 woman resided in a household, assessors asked 1 woman to participate. Sixteen assessors were given 4 days of training in assessment instruments and specific questions pertaining to exclusion criteria (e.g., suicide risk, psychosis, indications of cognitive impairment). Assessors were also instructed in psychological first aid (PFA) to provide the assessors with basic, non-intrusive skills to respond to acute distress. Following informed written consent, participants completed the 12-item General Health Questionnaire (GHQ-12 [21]; a measure that identifies psychological distress) and the WHO Disability Assessment Schedule (WHODAS) version 2.0 [22] (a measure of functioning). Inclusion criteria were a history of GBV, score of 3 or above on the GHQ-12 (using the dichotomous scoring method; range 0–12), and a score of 17 or above on WHODAS. Screening cutoffs were used to identify participants dichotomously (e.g., screening either positive or negative for distress), but this scoring method is less sensitive to treatment outcomes that aim to measure change in severity. Using the full range of Likert-scale measures is much more sensitive to change and hence was used here as the primary outcome. When used as screener, the GHQ-12 is scored dichotomously, with total score ranging 0–12, and a cutoff of 3 or higher can be used to indicate elevated distress [23]. The WHODAS cutoff of 17 was employed because this identifies the 90th percentile of impaired functioning across populations in 10 countries [24]; we used this inclusion criterion to ensure that we recruited women with impairment associated with their distress. The inclusion criterion of GBV was endorsement of any (prior or current) experience of interpersonal violence on either the Life Events Checklist (LEC [25]) or the WHO Violence Against Women Instrument (WHO-VAW [26]), which were administered at baseline assessment. Exclusion criteria included (a) imminent plans of suicide, (b) psychotic disorders, or (c) severe cognitive impairment. Assessors referred any cases of threat of harm or self-harm to local services. Assessment and PM+ sessions were conducted on an individual basis to maintain the safety and anonymity of participants.

### Randomisation and masking

Participants were randomly allocated (on a 1:1 ratio) to either a 5-week course of PM+ or EUC. Women were instructed that they would receive either 5 sessions with a CHW who would teach them skills to help them cope with stress or referral to a community nurse who would provide counselling for their problems. Randomisation was conducted at the University of New South Wales, Australia, by staff who were independent of the trial using computerised software that generated random number sequences. Assessors were blind to treatment condition, and blindness was optimised by having assessors trained and managed separately from CHWs. Fidelity of masking was measured by having assessors guess the condition of each participant at the end of each assessment. Assessors correctly guessed the condition of participants at a chance rate at both posttreatment (50.6%) and follow-up (47.5%), indicating that blindness was maintained.

### Procedures

Prior to the trial, the translation and cultural adaptation of PM+ was reviewed in 2 workshops with experts on PM+, translators, and CHWs to ensure that the assessment tools and intervention were appropriate in the Nairobi context. The measures and intervention were adapted for cultural appropriateness in terms of language, metaphors, content, concepts, goals, methods, and context [27].

Following screening for distress and impaired functioning, eligible participants who agreed to participate were administered the baseline assessment battery by an independent assessor. Participants were reimbursed KSh 300 (approximately US$3) for each assessment. Participants allocated to PM+ were offered 5 weekly 90-minute individual sessions (the full English and Swahili versions of the manual are available at http://www.who.int/mental_health/emergencies/problem_management_plus/en/).

### Interventions

The PM+ sessions were provided in the participants’ home, unless they preferred to do them in an alternate location for safety or privacy reasons. If a session was missed, the CHW telephoned the participant to reschedule the appointment; the participant was regarded as not continuing after 3 failed attempts to reschedule. PM+ commenced with an introduction to the program, motivational interviewing, psychoeducation, and stress management (Session 1); problem-solving strategies focused on specific problems nominated by the participant and review of stress management strategies (Session 2); behavioural activation and review of problem-solving and stress management (Session 3); strengthening social supports and review of stress management, problem-solving, behavioural activation, and social supports (Session 4); and reinforcement of all strategies and relapse prevention education (Session 5). Participants allocated to EUC were referred to primary healthcare centres, where nurses provided non-specific counselling. The nurses providing EUC did not follow a specific manual, they could use the strategies and number of sessions they deemed appropriate, and each nurse could use their judgement on rescheduling missed appointments with participants; there was continuity of the same nurse and same clinic for each woman in EUC.

Twenty-three CHWs were engaged to provide PM+. The CHWs had 10 years’ school education and did not have prior training or experience in mental healthcare. The CHWs were provided with a 64-hour training program (delivered by KSD) over 8 days, which is comparable to training given to non-specialist health workers to deliver other psychological interventions [10,28,29]. Two local supervisors who were experienced psychologists were also trained in PM+. Training covered knowledge of common mental health conditions, basic counselling delivery, PM+, and self-care strategies. CHWs also received a 1-day training in PFA to prepare them for managing people in crisis (e.g., ongoing violence) who required immediate attention and possible referral. Training also addressed issues related to GBV, as well as ethical and confidentiality matters. Each CHW delivered PM+ to approximately 3 clients under local supervision, after which CHWs were assessed for competency based on the supervisor’s evaluation of mock interviews; supervisors were trained to use a standardised rating scale based on established competency scales [30] to assess for key strategies required of CHWs, such as verbal and non-verbal communication, rapport, and clarity of teaching of PM+ strategies. Three CHWs failed to pass competency assessments and did not participate in the trial. During the trial, CHWs received 2 hours of weekly supervision by the local supervisor, who provided the supervision in 4 separate groups to the CHWs (5 CHWs per group). The local supervisors received 1.5 hours of weekly training and mentoring in supervision by KSD via Skype. To ensure continuity between sessions, each participant in the trial was seen by the same CHW for each PM+ session; CHWs provided PM+ to between 8 and 12 women each. EUC was provided by 6 community nurses at clinics in the area. These nurses were selected because they routinely provided health services in the local clinics, and each had 14 years’ education, including a diploma level of education, and at least several years of experience in counselling HIV patients. These nurses were provided with manualised 2-day non-specific training in counselling skills [31] and PFA [32]. Usual psychosocial care in Kenya for people identified with significant psychological distress tends to involve untrained health workers providing advice. In contrast, this study used trained nurses in the EUC group to provide an enhanced level of care for the participants who were not randomised to PM+. The nurses did not receive supervision. They completed monitoring forms in which they recorded when a participant sought help for mental health issues and the strategies they provided in each session.

To assess protocol adherence, 10% of randomly selected PM+ sessions were attended by a supervisor who used a checklist to ensure relevant treatment elements were provided. Adverse reactions were monitored and recorded throughout screening and the intervention. Indications of psychiatric crisis (e.g., imminent suicidal risk as defined by suicidal plan) or need for acute protection were referred to the local advisory board, and referral to appropriate services was made (including local hospitals providing psychiatric care).

### Measures and outcomes

#### Psychological distress

The primary outcome was the GHQ-12, which indexes psychological distress, including anxiety and depression, “in the past few weeks”. The GHQ-12 comprises 12 questions scored on a 4-point scale ranging from 0 to 3 (range 0–36; higher scores indicate more severe psychological distress). The GHQ-12 has been widely used across LMICs, including a Kiswahili version in Kenya [33]. A cutoff of 12/13 (using the 4-point continuous scoring system rather than the dichotomous scoring system used for screening) can be used to detect psychological morbidity, as has previously been used in Kenya [34]. The internal consistency for the GHQ-12 in this sample was 0.78.

#### Functioning

Functional impairment was measured with the WHODAS 2.0. This 12-item instrument assesses difficulty in completing activities covering cognition, mobility, self-care, socialising, and life activities in the last 30 days (range 0–48; higher scores indicate greater impairment severity). The WHODAS 2.0 has been used widely as a screening and outcome measure, demonstrates moderate to strong evidence of construct- and criterion-related validity, displays good sensitivity to change [22], and has also been used previously in Kenya [35]. The internal consistency for the WHODAS in this sample was 0.66.

#### Posttraumatic stress

The Posttraumatic Stress Disorder Checklist (PCL) for DSM-5–Civilian Version (PCL-5 [36]) was used to measure the 20 symptoms of PTSD according to DSM-5 (range 0–80; higher scores indicate greater severity). The PCL-5 was adapted to ask for symptoms in the last week (rather than month) to enhance sensitivity to change (although psychometric studies are based on the 1-month reporting period). The internal consistency for the PCL-5 in this sample was 0.92.

#### Personalised outcomes

The Psychological Outcome Profiles (PSYCHLOPS) scale is used to assess change in relation to problems that are identified by the participant [37]. It asks respondents to nominate 2 of their main problems and to provide ratings on the magnitude of the problems and their effects on functioning and well-being over the previous week. It is sensitive to change, and is internally reliable [37]. PSYCHLOPS assesses the impact of interventions on problems that are not necessarily assessed by other standardised measures, and this can be useful in the context of LMICs, where people can suffer a diverse range of problems. The internal consistency for PSYCHLOPS in this sample was 0.64.

#### Stressful life events

The LEC [25] was used to assess exposure to traumatic events over the participant’s lifetime. The measure indexes 15 events, including rape. A previously used Kiswahili version of the assessment was applied [38]. The LEC was used (a) to profile the population by indexing level of trauma exposure, (b) to identify GBV, and (c) as an outcome measure to determine if the interventions had a differential impact on trauma exposure between treatment and follow-up. At the follow-up assessment, the LEC was asked in relation to events that occurred since the last assessment. The internal consistency for the LEC in this sample was 0.77.

#### Gender-based violence

Five key questions from WHO-VAW [26] were included in the assessment. Women were asked to indicate the frequency of different types of physical and sexual violence they had experienced by an intimate partner or other adult since the age of 15 years. Experience of GBV was defined in this cohort as prior or current actual or threatened sexual or nonsexual violence committed against a woman, as reported on either the LEC or WHO-VAW; that is, GBV encompassed both intimate partner violence (IPV) and GBV from people other than intimate partners.

#### Health service use

To address the study’s hypothesis that PM+ may reduce the need to utilise health services as a result of improved mental health, the following self-reported items from the Service Receipt Inventory (SRI) [39] were selected: (a) hospital in-patient admission, (b) number of hospital out-patient consultations, (c) amount of medication use, and (d) traditional healing consultation.

All measures were administered at baseline, and the GHQ-12, PCL-5, PSYCHLOPS, and WHODAS were assessed at posttreatment (7 weeks after baseline assessment) and 3 months after treatment (18 weeks after baseline). The LEC and SRI items were assessed at 3 months. The 3-month assessment was used as the primary outcome in order to determine the medium-term effects of the PM+ intervention.

### Statistical analyses

The sample size was calculated on the prediction of a small to moderate effect size (*d* = 0.4) on the GHQ-12 at the 3-month follow-up assessment (based on prior interventions in primary care [40]). Power calculations indicated a minimum sample size of 133 participants per group using the test for paired means (power = 0.95, alpha = 0.05, 2-sided). On the basis of 30% attrition at follow-up, it was estimated that 346 participants (173 per group) were needed. It was estimated that 70% of women in the distressed and impaired sample would have a history of GBV, and so it was intended to include at least 494 women in the study to arrive at the required sample size of women with a history of GBV.

Analyses focused primarily on intent-to-treat analysis. Using SPSS version 24, hierarchical linear models (HLMs) were used to study the differential effects of each treatment condition because this method allows the number of observations to vary between participants and handles missing data by calculating estimates of trajectories using maximum likelihood estimation [41]. Fixed effects were tested for intervention condition and time of assessment. Random effects (including CHW/community nurse and assessment point) in the unstructured models provided an index of the relative effects of the treatments over time. Fixed effects parameters were tested with the Wald test (*t* test, *P* < 0.05, 2-sided) and 95% confidence intervals. Analyses focus on the primary (GHQ-12) and secondary (WHODAS, PCL, PSYCHLOPS) outcomes between PM+ and EUC, with the main outcome point being the 3-month follow-up relative to baseline. All results are based on estimated mean values derived from HLM analyses, except for dichotomous categorical outcomes that are limited to treatment completers. Estimated mean differences are reported for the estimated follow-up score subtracted from the baseline score on each outcome measure. Effect sizes are determined by calculating the difference between the estimated means divided by the raw standard deviation. Service utilisation (based on SRI responses) and the proportion of women who no longer scored above the GHQ-12 cutoff for psychological morbidity (among treatment completers) were calculated for those who completed the 3-month follow-up. These findings focus on women who met the inclusion criterion of being exposed to GBV (81% of all women who screened positive on high distress and impaired functioning). The data analyses followed an a priori statistical analysis plan (S1 Text).

## Results

**Fig 1:**
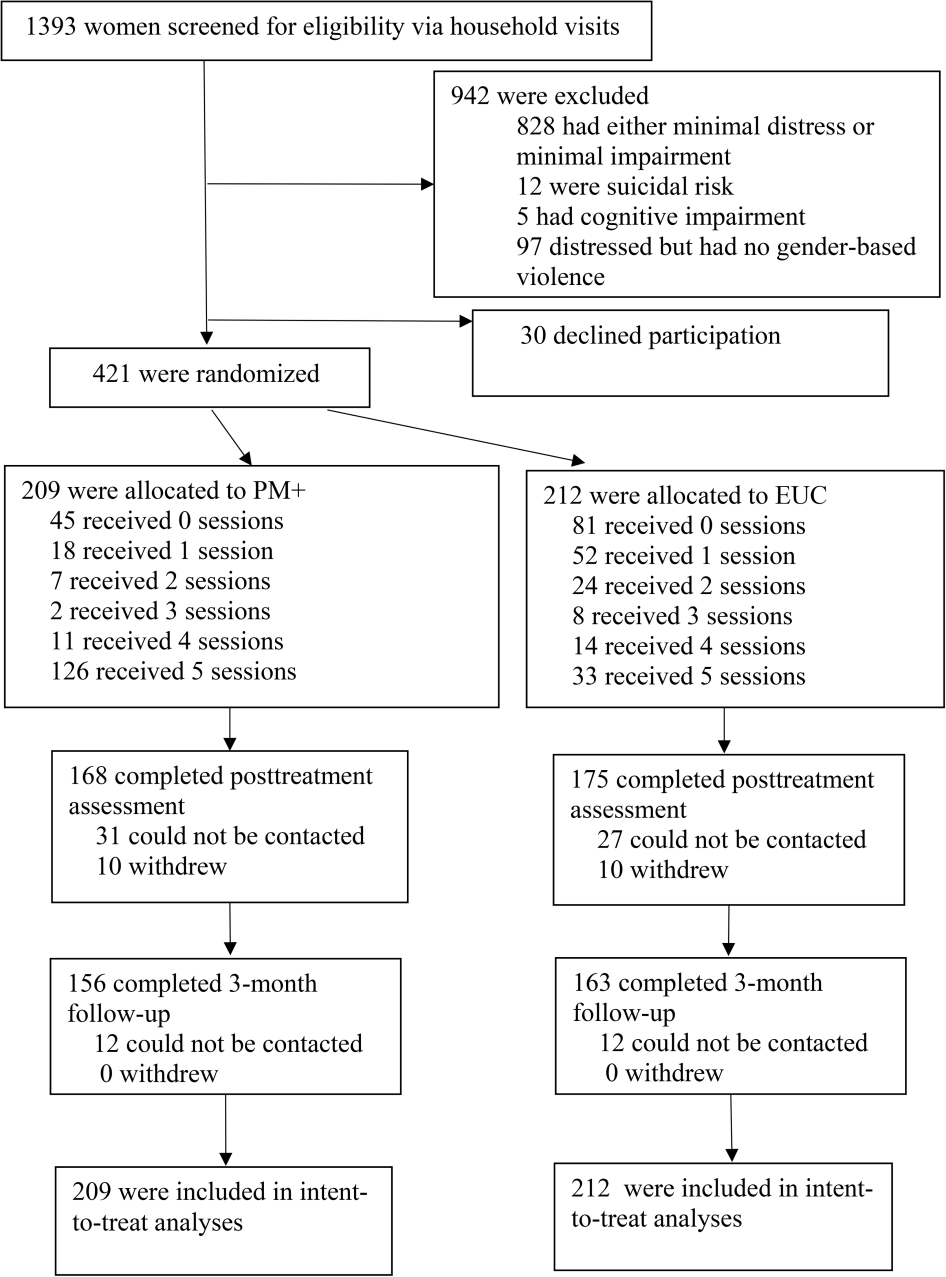
Flow diagram of progress through phases of a randomised trial comparing problem management plus versus enhanced usual care among women with a history of gender-based violence in urban Kenya. EUC, enhanced usual care; PM+, Problem Management Plus.

Between 15 April 2015 and 20 August 2015 (with final follow-up assessments completed on 16 January 2016), 1,393 women were interviewed, and 518 (37%) screened positive for distress and impairment, of whom 421 (81%) reported a history of GBV (prior or current) and satisfied the inclusion criteria cutoffs on the GHQ-12 and WHODAS. The rate of GBV in the screened sample was higher than predicted (81% versus 70%), and accordingly the sample size was larger than planned. There were 209 women randomised to PM+, and 212 to EUC. To comply with ethical concerns about not publicly singling out women who had experienced GBV, all women who were identified during the screening as distressed and functionally impaired (irrespective of GBV history) were randomised to one of the treatment groups(results for the entire sample are reported in S3 Text). Despite the randomisation of all women meeting the criteria for distress and impairment, the intended focus of the study was the impact of PM+ of women who had experienced GBV. The flowchart of participant recruitment and retention is reported in Fig 1. Sample characteristics are presented in Table 1.

**Table 1:** Participant characteristics and trauma exposure assessed at baseline.

Characteristic or exposure	PM+ (*n* = 209)	EUC (*n* = 212)	*t* (*P* value)
**Age, mean (SD)**	35.2 (14.1)	35.9 (12.7)	0.57 (0.57)
**Education, mean (SD)**	8.7 (3.6)	8.2 (4.2)	1.20 (0.23)
**Marital status, *n* (%)**			0.68* (0.98)
Single	25 (12.0)	30 (14.1)	
Married	122 (58.4)	119 (56.1)	
Divorced/separated	42 (20.1)	45 (21.2)	
Widowed	20 (9.5)	18 (8.5)	
**Working, *n* (%)**	104 (49.8)	108 (50.9)	0.12* (0.73)
**Suicidal intention in past month, *n* (%)**	50 (23.9)	35 (16.5)	2.80* (0.09)
**LEC total, mean (SD)**	7.0 (3.2)	6.7 (3.3)	0.75 (0.45)
**LEC event, *n* (%)**			
Disaster	118 (56.5)	102 (48.1)	
Fire	123 (58.8)	116 (54.7)	
Road accident	121 (57.9)	110 (51.9)	
Serious accident	97 (46.4)	105 (49.5)	
Chemical exposure	70 (33.5)	70 (33.0)	
Physical assault	155 (74.2)	153 (72.2)	
Assault with weapon	104 (49.8)	95 (44.8)	
Sexual assault	59 (28.2)	72 (34.0)	
Unwanted sexual contact	59 (28.2)	63 (29.7)	
War exposure	59 (28.2)	59 (27.8)	
Kidnapped	43 (20.6)	38 (17.9)	
Life-threatening illness	109 (52.1)	103 (48.6)	
Witness violent death	103 (49.3)	98 (46.2)	
Unexpected death of loved one	159 (76.1)	157 (74.1)	
Intimate partner violence	153 (73.2)	152 (71.7)	
**Baseline score, mean (SD)**			
GHQ-12	19.1 (6.0)	18.8 (5.9)	0.39 (0.69)
PCL	33.7 (19.7)	31.5 (18.9)	1.2 (0.24)
WHODAS	28.0 (7.5)	27.2 (7.2)	1.1 (0.26)
PSYCHLOPS	16.6 (3.2)	16.4 (3.3)	0.67 (0.50)

*Chi square test.

EUC, enhanced usual care; GHQ-12, 12-item General Health Questionnaire (range 0–36; higher scores indicate elevated anxiety or depression); LEC, Life Events Checklist; PCL, Posttraumatic Stress Disorder Checklist (range 0–80; higher scores indicate greater severity); PM+, Problem Management Plus; PSYCHLOPS, Personalized Outcome Profiles (range 0–20; higher scores indicate poorer outcome); WHODAS, WHO Disability Adjustment Scale (range 0–48; higher scores indicate more severe impairment).

Planned comparisons of women in the PM+ and EUC groups indicated that these 2 groups did not differ on any pretreatment factors. Among the 421 women with a history of GBV (mean age 35.56 y [SD 13.39]) enrolled in the study, 319 (75.77%) completed the 3-month follow-up. The level of attrition was within the 30% margin with which the power analysis was calculated. Sensitivity analyses indicated that the results were not biased by the attrition of the sample. Women who were retained at follow-up did not differ from those who were lost to follow-up in terms of age, education level, or baseline score on any outcome measure. Further, analyses including only completers at follow-up yielded comparable findings to the intent-to-treat analyses. Pretreatment measures were also compared between participants who completed all 5 sessions of PM+ and those who did not; completers did not differ on any baseline features except for PCL score, for which women who completed all 5 sessions reported more severe PTSD at baseline than those who did not complete all 5 sessions (mean baseline PCL score: 35.28 [SD 20.13] versus 31.32 [SD 18.95], mean difference = 3.96 [95% CI −7.12 to 0.80], *P* = 0.01). More women in the EUC than PM+ group did not attend any sessions (81 [38.2%] versus 45 [21.5%], odds ratio [OR] 3.24 [95% CI 2.04–5.03)], *P* = 0.001).

Table 1 illustrates that approximately three-quarters of the sample reported a history of being physically assaulted, one-half a history of being assaulted with a weapon, one-half a history of witnessing a homicide or violent death, and nearly one-half a history of sexual assault (see Table 2 for further details on IPV).

**Table 2:** Violence against women assessed at baseline.

Event	PM+ (*n* = 209)	EUC (*n* = 212)	χ^2^ (*P* value)
**Assaulted**			
Ever	145 (69.4)	150 (70.7)	1.21 (0.27)
In past year	80 (38.3)	86 (40.6)	0.49 (0.48)
**Choked/burnt**			
Ever	37 (17.7)	43 (20.3)	0.54 (0.46)
In past year	17 (8.1)	18 (8.5)	1.05 (0.32)
**Assaulted with weapon**			
Ever	50 (23.9)	57 (26.9)	0.65 (0.42)
In past year	26 (12.4)	28 (13.2)	0.09 (0.77)
**Forced sex**			
Ever	74 (35.4)	59 (27.8)	1.95 (0.16)
In past year	46 (22.0)	35 (16.5)	0.23 (0.23)

Data given as n (percent).

EUC, enhanced usual care; PM+, Problem Management Plus.

There were 12 reported adverse events, which all involved marked suicidal risks that were detected during screening for distress before randomisation; these women were referred for immediate assistance and not enrolled in the study. No reported adverse effects occurred during treatment.

In terms of the problems reported by women on PSYCHLOPS, the major problems pertained to financial concerns (51.78%), health issues (20.19%), and problems with husbands/partners (15.20%).

In terms of EUC, 131 (61.8%) participants sought assistance from a community nurse, attending a median of 1.0 (*M* = 2.1, SD = 1.8) visit. In terms of the strategies reported by the community nurses in EUC, 65.57% reported non-specific counselling, 26.89% provided psychosocial advice, 7.00% encouraged activity, 7.08% encouraged social support, and 3.30% instructed in coping strategies. This suggests that only 3.30% to 7.08% of strategies reported by nurses delivering EUC overlapped with content contained in PM+.

The fidelity checks indicated that CHWs adhered to the protocol by addressing the requisite PM+ components in the appropriate sessions, including stress reduction (91.39%), problem-solving (94.25%), behavioural activation (83.25%), and accessing social support (92.82%).

### Primary outcome

**Fig 2:**
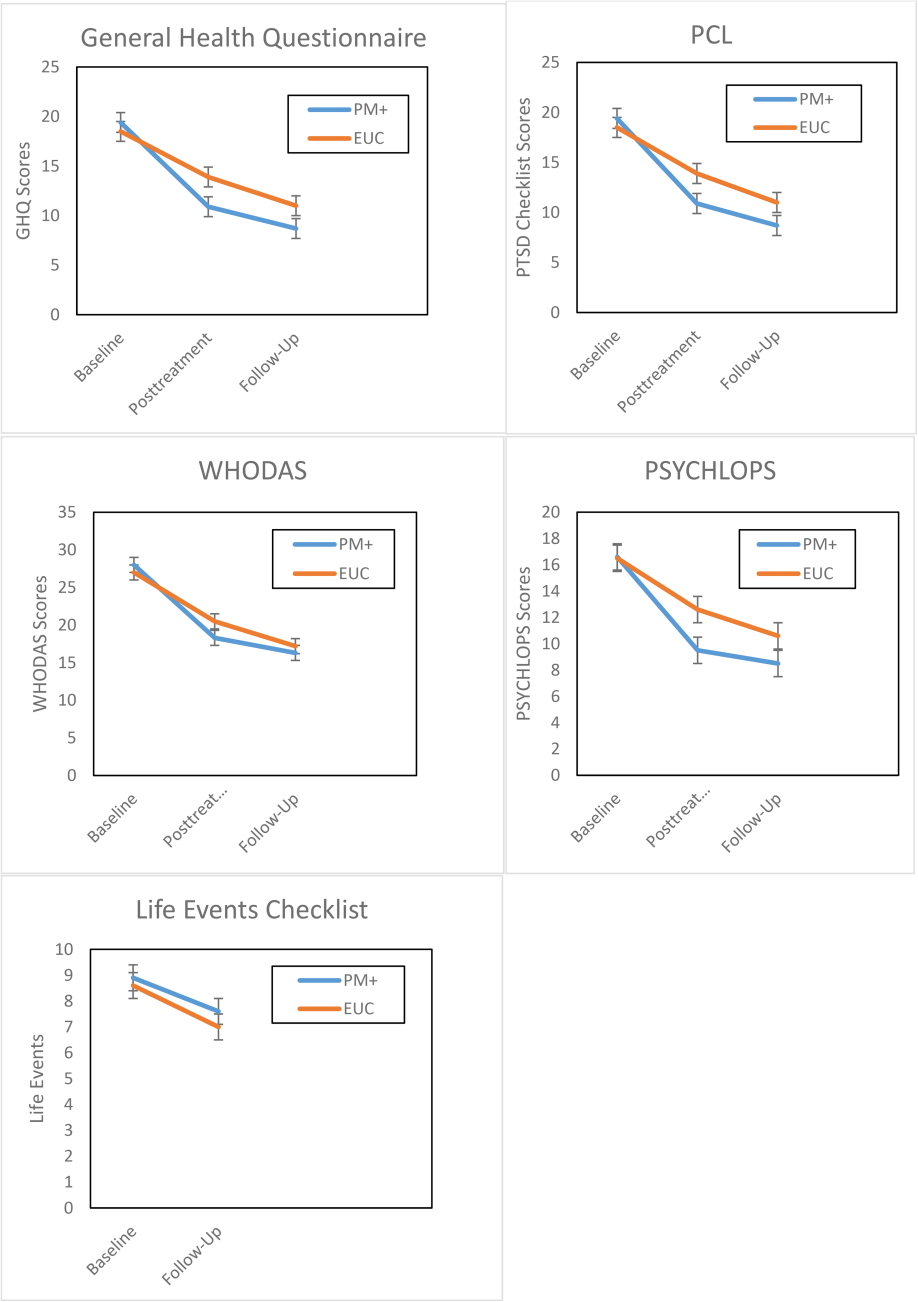
Estimated means of primary and secondary outcomes. Values based on estimated means derived from hierarchical linear model analyses. Error bars indicate 95% confidence intervals. EUC, enhanced usual care; GHQ, General Health Questionnaire; PCL, Posttraumatic Stress Disorder Checklist; PM+, Problem Management Plus; PSYCHLOPS, Personalized Outcome Profiles; WHODAS, WHO Disability Adjustment Scale.

Table 3 provides the estimated mean scores for the GHQ-12. Both treatment groups displayed marked reductions in severity of psychological distress over time (see Fig 2). The between-group difference at 3-month follow-up (relative to baseline) was 3.33 (95% CI 1.86–4.79, *P* = 0.001), indicating that PM+ led to greater medium-term reductions in psychological distress than EUC. Fewer women in the PM+ than the EUC group met the criterion for psychological morbidity on the GHQ-12 at posttreatment (34.52% versus 59.42%, OR 2.8 [95% CI 2.1–3.6], *P* < 0.001) and follow-up (21.15% versus 38.65%, OR 2.3 [95% CI 1.7–3.0], *P* < 0.001). In terms of group difference in reduction of GHQ-12 score from baseline to 3-month follow-up, there was a moderate effect size in favour of PM+ (0.57, 95% CI 0.32 to 0.83) (see Table 3). There was a significant positive correlation between number of PM+ sessions attended and reduction in GHQ-12 score at 3 months relative to baseline (*r* = 0.24, *P* = 0.004).

**Table 3:** Estimated mean scores for primary and secondary outcome measures at baseline, posttreatment, and 3-month follow-up for women with a history of gender-based violence.

Category	Outcome	PM+ (*n* = 209)	Enhanced usual care (*n* = 212)	Estimated mean difference from baseline	*P* value	Effect size (95% CI)
**Primary outcome**	**GHQ-12**					
	Baseline, mean (95% CI)	19.4 (18.5–20.3)	18.5 (17.6–19.4)			
	Posttreatment, mean (95% CI)	10.9 (9.9–12.0)	13.9 (12.9–15.0)	3.91 (2.40–5.42)	0.001	0.67 (0.41 to 0.93)
	3-month follow-up, mean (95% CI)	8.7 (7.6–9.7)	11.0 (10.0–12.1)	3.33 (1.86–4.79)	0.001	0.57 (0.32 to 0.83)
**Secondary outcomes: continuous**	**PCL**					
	Baseline, mean (95% CI)	33.9 (30.9–36.9)	31.5 (28.5–34.5)			
	Posttreatment, mean (95% CI)	9.7 (7.1–12.3)	14.4 (11.8–17.0)	7.13 (3.22–11.03)	0.001	0.37 (0.17 to 1.03)
	3-month follow-up, mean (95% CI)	6.6 (4.4–8.8)	8.2 (4.4–8.8)	3.95 (0.06–7.83)	0.05	0.26 (0.02 to 0.50)
	**WHODAS**					
	Baseline, mean (95% CI)	28.1 (26.0–28.2)	27.0 (25.9–28.1)			
	Posttreatment, mean (95% CI)	18.3 (17.1–19.5)	20.5 (19.3–21.7)	3.26 (1.49–5.03)	0.001	0.44 (0.20 to 0.68)
	3-month follow-up, mean (95% CI)	16.3 (15.1–17.4)	17.2 (16.1–18.2)	1.96 (0.21–3.71)	0.03	0.21 (0.00 to 0.41)
	**PSYCHLOPS**					
	Baseline, mean (95% CI)	16.6 (16.0–17.2)	16.5 (15.9–17.1)			
	Posttreatment, mean (95% CI)	9.5 (8.6–10.4)	12.6 (11.7–13.5)	3.20 (2.09–4.32)	0.001	1.00 (0.65 to 1.35)
	3-month follow-up, mean (95% CI)	8.5 (7.6 to 9.5)	10.6 (9.6–11.5)	2.15 (0.98–3.32)	0.001	0.67 (0.31 to 1.03)
	**Life Events Checklist**					
	Baseline, mean (95% CI)	8.9 (8.4–9.5)	8.6 (8.1 to 9.2)			
	3-month follow-up, mean (95% CI)	7.6 (7.0–8.3)	7.0 (6.4–7.7)	0.31 (0.02–1.23)	0.51	0.03 (−0.23 to 0.15)
**Secondary outcomes: categorical**	**Psychological morbidity based on GHQ-12**					
	Baseline, *n*/total (%)	178/209 (85.2)	182/212 (85.8)		0.36	1.3 (0.7 to 2.2)
	Posttreatment, *n*/total (%)	60/168 (35.7)	102/175 (58.3)		<0.001	2.5 (1.6 to 3.9)
	3-month follow-up, *n*/total (%)	39/156 (25.0)	59/163 (36.2)		<0.03	1.7 (1.0 to 2.8)
	**PTSD diagnosis based on PCL**					
	Baseline, *n*/total (%)	153/209 (73.2)	151/212 (71.2)		0.89	1.03 (0.7 to 1.6)
	Posttreatment, *n*/total (%)	40/164 (24.4)	51/172 (29.7)		0.28	1.31 (0.8 to 2.1)
	3-month follow-up, *n*/total (%)	28/155 (18.1)	24/163 (14.7)		0.41	0.78 (0.4 to 1.4)

P values for continuous measures refer to between-group differences in change from baseline. P values for categorical measures refer to between-group differences at each assessment. Continuous outcomes are based on estimated mean values derived from HLM analyses. Categorical outcomes are based on treatment completers. Calculated mean differences differ marginally from absolute differences between estimated means because the estimated mean differences are derived from HLMs.

GHQ-12, 12-item General Health Questionnaire (range 0–36; higher scores indicate elevated anxiety or depression); HLM, hierarchical linear model; PCL, Posttraumatic Stress Disorder Checklist (range 0–80; higher scores indicate greater severity); PM+, Problem Management Plus; PSYCHLOPS, Personalized Outcome Profiles (range 0–20; higher scores indicate poorer outcome); PTSD, posttraumatic stress disorder; WHODAS, WHO Disability Adjustment Scale (range 0–48; higher scores indicate more severe impairment).

### Secondary outcomes

PCL, WHODAS, PSYCHLOPS, and LEC scores are also displayed in Table 3 and Fig 2. In terms of impaired functioning, the between-group difference at 3-month follow-up on the WHODAS was 1.96 (95% CI 0.21–3.71, *P* = 0.03), indicating that PM+ led to greater reductions in functional impairment than EUC. The between-group effect size at follow-up was small (0.26, 95% CI 0.02–0.50).

In terms of PTSD, the between-group difference at 3-month follow-up was 3.95 (95% CI 0.06–7.83, *P* = 0.05), indicating that PM+ led to greater reductions in PTSD symptoms than EUC. The small between-group effect size (0.21, 95% CI 0.00–0.41) suggests a marginal difference between groups regarding PTSD reduction. Comparable numbers of women in the PM+ and EUC groups met PTSD diagnosis criteria at follow-up (18.1% versus 14.7%, OR 0.78 [95% CI 0.40–1.40], *P* = 0.41).

Regarding personalised outcomes as measured by PSYCHLOPS, the between-group difference at 3-month follow-up was 2.15 (95% CI 0.98–3.32, *P* = 0.001), indicating that PM+ led to a greater reduction in personally identified problems than EUC. At follow-up, there was a moderate between-group effect size in favour of PM+ (0.67, 95% CI 0.31–1.03).

In terms of scores on exposure to life stressors, the between-group difference at 3-month follow-up was 0.31 (95% CI 0.02–1.23, *P* = 0.51), indicating no difference in exposure to stressful life events during the period of the study between the 2 groups. There was a very small between-group effect size (0.03, 95% CI −0.23 to 0.15).

Regarding health service utilisation, there were no differences between the PM+ and EUC groups at the 3-month follow-up on whether there had been a hospital admission (2.56% versus 3.07%, OR 1.13 [95% CI 0.52–2.45], *P* = 0.75), the number of out-patient consultations (*M* = 1.62, SD = 2.56, versus *M* = 1.58, SD = 2.43; mean difference −0.03 [95% CI −0.59 to 0.52], *P* = 0.90), medication use (*M* = 1.73, SD = 2.52, versus *M* = 2.02, SD = 3.49; mean difference 0.29 [95% CI −0.54 to 0.98], *P* = 0.42), or traditional healer engagement [*M* = 0.05, SD = 0.31, versus *M =* 0.16, SD = 0.88; mean difference 0.12 [95% CI −0.03 to 0.27], *P* = 0.12).

## Discussion

This study indicates that PM+ delivered by lay CHWs moderately reduced psychological distress relative to EUC. This finding reinforces evidence that effective interventions can be delivered using supervised non-specialised workers [10,28,42]. A major advance of this trial is that it shows that a brief behavioural intervention comprising a maximum of 5 sessions can reduce psychological morbidity among women living in urban poverty with a history of GBV. This program was safe insofar as it did not cause adverse outcomes, and could be used by women who may be experiencing current GBV, in combination with relevant protective interventions. Informed by this and another large trial [19], WHO has decided to publish the individual protocol of PM+ on its website as a vehicle for dissemination [14]. When one considers the utility of PM+ in comparison to other interventions with proven efficacy for women who have experienced GBV [10], the current findings indicate that it may be a viable program as well, and may be particularly applicable in settings where resources for supervision or the capacity of recipients restrict the number of sessions that can be offered. Further study is required to compare PM+ versus longer versions of psychological treatments to determine their relative feasibility, affordability, and cost-effectiveness. Long-term implementation studies are needed to evaluate the sustainability of PM+ in the community as PM+, like any other community intervention, requires an appropriately resourced community workforce.

A key finding was that distressed women who had experienced GBV could be detected via screening for distress and impairment. Among women who screened positively for psychological distress and impaired functioning, 81% reported a history of GBV. International guidelines call for integrating care for women affected by GBV into general services as a means of reducing the obstacles created by the stigma of being identified as a victim of GBV [5], and thereby promoting greater reach to more affected women in a safe manner [7]. General health services may be complemented with PM+, thereby reaching many more GBV-affected women without the problems associated with GBV-related social stigma.

We note that PM+ and EUC did not differ in their effects on either stressful life events or health service utilisation at follow-up. There is evidence that a psychosocial intervention reduced the occurrence of IPV in a program that was targeted to both men and women [43]. It is possible that we did not observe a difference in the occurrence of stressful life events in the current study because we focused exclusively on women; there is a need to determine in future studies if PM+ can reduce GBV when delivered to males as well females. We also note that the assessment of health service utilisation was minimal and reliant on self-report in this study, and more objective measures of health service utilisation in future studies are needed.

### Generalisability of the findings

These findings point to future directions for evaluating mental health provisions for women affected by GBV. A major hurdle in scaling up mental health interventions is the extent to which they can be affordably adopted by local services [4]. The brevity of the PM+ program enables brief and effective intervention that may be supplemented by more resource-intensive strategies for those who need more than PM+. It is worth noting that PM+ had less impact on PTSD symptoms than on general psychological distress, and this effect can be contrasted to a previous trial of psychotherapy in individuals who had experienced sexual violence in Congo that reported a large effect size in relation to PTSD symptom reduction [10]; it is possible that this difference between studies occurred because PM+ did not include emotional processing strategies, which are foundational in many PTSD-focused treatments [10,28,44]. Moreover, the current findings suggest that having more resource-intensive interventions additionally available may be useful because 25% of the women who completed the 3-month assessment still met the criterion for psychological morbidity, suggesting they required further mental health assistance. This interpretation is supported by the finding that women who completed all sessions of PM+ had more severe initial PTSD, raising the possibility that they had greater need for longer or more intensive intervention. Given the scarcity of resources in many regions affected by GBV, there is a need for implementation trials that evaluate PM+ within a stepped-care framework in LMICs. There is a need for full cost-effectiveness analysis of the PM+ intervention that takes into account the costs of training, supervision, and lay CHWs relative to the benefits gained in terms of improved mental health.

### Limitations

A series of limitations are noted. First, this study was limited to women, and so it is unknown if comparable effects would be achieved with men. Second, since assessors were trained in PFA, it is possible, though unlikely, that this training led to improvements in all participants. Third, accurate assessment of session duration was not obtained. Fourth, assessments relied on self-reported data rather than structured interviews, and longer-term follow-up assessments would have been preferable because treatment effects can subside over time [45]. It is critical that future replications of this intervention assess participants at longer time frames to determine the sustainability of treatment effects. Fifth, the measures employed in the study do not fully index local manifestations of distress or functioning. Sixth, participants in the PM+ and EUC groups differed on a number of variables, including the number of sessions, the pre-existing educational levels of providers, the nature of the training given to providers, the context of service delivery, and the role of supervision. Moreover, although nurses kept records of the number of sessions completed, they did not record this information for each participant; this precluded analysis of EUC effects according to number of sessions attended. Accordingly, we cannot exclude the possibility that the greater symptom reduction in the PM+ group may be attributed to one or more of these differences between the PM+ and EUC conditions. Seventh, we did not measure mechanism-related processes because of the priority of indexing the effectiveness of PM+ and limiting the assessment burden on participants. Eighth, use of supervisors’ checks on protocol adherence is not ideal because the presence of supervisors may have influenced CHWs’ performance; however, recording of sessions was not possible because of participants’ concerns about confidentiality. Moreover, optimal assessment of adherence would have used more than the 10% of sessions measured in this study. Finally, participants’ characteristics could not be compared to population data, and accordingly the representativeness of the sample cannot be determined. These limitations are offset by strengths that include the relevance of the research question, the maintained blindness at each assessment, good retention in both groups for the follow-up assessment, effective randomisation to conditions, adherence to the treatment protocol, and the open-access availability of the intervention manual on the WHO website as a result of this positive evaluation.

An unexpected finding was that participants in EUC markedly improved on every symptom measure. It is worth noting that the nurses providing the EUC were substantially educated, with professional nursing qualifications as well as additional training and experience in HIV counselling. In this sense, the nurses providing the EUC were much better qualified (14 years of education) than the CHWs who provided PM+ (10 years of education). This may have led to the comparator condition being an excessively stringent test for PM+. Relatedly, the power analysis was determined on the basis of a smaller reduction in GHQ-12 scores in EUC participants than was observed, and this may have reduced the resulting between-condition effect size. We also note that we did not include an attention control condition in this study, and accordingly we cannot exclude the possibility that receiving home visits (without the PM+ strategies) may have contributed to the symptom reduction. In adopting an EUC condition in this trial, we addressed the imperative of providing services to women in need [46]. Finally, we note that the relatively few sessions that women attended with the EUC nurses raises the possibility that symptom reduction occurred as a result of regression to the mean over time or repeated assessments.

### Conclusion

A brief lay-administered psychological intervention based on behavioural therapy techniques led to moderate reductions in psychological distress after 3 months in women in community settings in peri-urban Kenya with a history of GBV. Whereas the effect of treatment was not as strong as previously reported in interventions requiring more sessions [10], the briefer intervention employed in this trial may be delivered at less cost and with fewer demands on recipients. These factors may promote implementation and scaling up of this program in poorly resourced settings.

## Supporting information

S1 Text Study protocol. (DOCX)

S2 Text CONSORT checklist. (DOC)

S3 Text Supplementary analyses. (DOCX)

S4 Text Ethics approval. (PDF)

## Abbreviations

CBT: cognitive behaviour therapy
CHW: community health worker
EUC: enhanced usual care
GBV: gender-based violence
GHQ-12: 12-item General Health Questionnaire
HLM: hierarchical linear model
IPV: intimate partner violence
LEC: Life Events Checklist
LMICs: low- and middle-income countries
OR: odds ratio
PCL: Posttraumatic Stress Disorder Checklist
PFA: psychological first aid
PM+: Problem Management Plus
PSYCHLOPS: Psychological Outcome Profiles
PTSD: posttraumatic stress disorder
SRI: Service Receipt Inventory
WHODAS: WHO Disability Adjustment Schedule
WHO-VAW: WHO Violence Against Women Instrument

## References

[1] World Health Organization. Responding to intimate partner violence and sexual violence against women: WHO clinical and policy guidelines. Geneva: World Health Organization; 2013. 24354041

[2] World Health Organization. Violence against women: a ‘global health problem of epidemic proportions’. Geneva: World Health Organization; 2013. Available from: http://www.who.int/mediacentre/news/releases/2013/violence_against_women_20130620/en/. Accessed: 2016 Dec 10.

[3] Heise LL, Kotsadam A. Cross-national and multilevel correlates of partner violence: an analysis of data from population-based surveys. Lancet Glob Health. 2015;3(6):e332–40. doi: 10.1016/S2214-109X(15)00013-3 26001577 10.1016/S2214-109X(15)00013-3

[4] Saxena S, Thornicroft G, Knapp M, Whiteford H. Resources for mental health: scarcity, inequity, and inefficiency. Lancet. 2007;370(9590):878–89. doi: 10.1016/S0140-6736(07)61239-2 17804062 10.1016/S0140-6736(07)61239-2

[5] Garcia-Moreno C, Hegarty K, d’Oliveira AF, Koziol-McLain J, Colombini M, Feder G. The health-systems response to violence against women. Lancet. 2015;385(9977):1567–79. doi: 10.1016/S0140-6736(14)61837-7 25467583 10.1016/S0140-6736(14)61837-7

[6] Oram S, Khalifeh H, Howard LM. Violence against women and mental health. Lancet Psychiatry. 2016;4(2):159–70. doi: 10.1016/S2215-0366(16)30261-9 27856393 10.1016/S2215-0366(16)30261-9

[7] Inter-Agency Standing Committee. Guidelines for integrating gender-based violence interventions in humanitarian action: reducing risk, promoting resilience and aiding recovery. 2015. Available from: http://gbvguidelines.org/. Accessed: 2016 Jan 5.

[8] Hegarty K, Tarzia L, Hooker L, Taft A. Interventions to support recovery after domestic and sexual violence in primary care. Int Rev Psychiatry. 2016;28(5):519–32. doi: 10.1080/09540261.2016.1210103 27686012 10.1080/09540261.2016.1210103

[9] Ertl V, Pfeiffer A, Schauer E, Elbert T, Neuner F. Community-implemented trauma therapy for former child soldiers in Northern Uganda: a randomized controlled trial. JAMA. 2011;306(5):503–12. doi: 10.1001/jama.2011.1060 21813428 10.1001/jama.2011.1060

[10] Bass JK, Annan J, McIvor Murray S, Kaysen D, Griffiths S, Cetinoglu T, et al Controlled trial of psychotherapy for Congolese survivors of sexual violence. N Engl J Med. 2013;368(23):2182–91. doi: 10.1056/NEJMoa1211853 23738545 10.1056/NEJMoa1211853

[11] Tirado-Munoz J, Gilchrist G, Farre M, Hegarty K, Torrens M. The efficacy of cognitive behavioural therapy and advocacy interventions for women who have experienced intimate partner violence: a systematic review and meta-analysis. Ann Med. 2014;46(8):567–86. doi: 10.3109/07853890.2014.941918 25211469 10.3109/07853890.2014.941918

[12] Rivas C, Ramsay J, Sadowski L, Davidson LL, Dunne D, Eldridge S, et al Advocacy interventions to reduce or eliminate violence and promote the physical and psychosocial well-being of women who experience intimate partner abuse. Cochrane Database Syst Rev. 2015;12:CD005043. 10.1002/14651858.CD005043.pub3 PMC9392211 26632986

[13] Dawson KS, Bryant RA, Harper M, Kuowei Tay A, Rahman A, Schafer A, et al Problem Management Plus (PM+): a WHO transdiagnostic psychological intervention for common mental health problems. World Psychiatry. 2015;14(3):354–7. doi: 10.1002/wps.20255 26407793 10.1002/wps.20255 PMC4592660

[14] World Health Organization. Problem Management Plus (PM+): individual psychological help for adults impaired by distress in communities exposed to adversity WHO generic field-trial version 1.0. Geneva: World Health Organization; 2016 Available from: http://www.who.int/mental_health/emergencies/problem_management_plus/en/. Accessed: 2017 Jul 18.

[15] Cuijpers P, van Straten A, Warmerdam L. Problem solving therapies for depression: a meta-analysis. Eur Psychiatry. 2007;22(1):9–15. doi: 10.1016/j.eurpsy.2006.11.001 17194572 10.1016/j.eurpsy.2006.11.001

[16] Cuijpers P, van Straten A, Warmerdam L. Behavioral activation treatments of depression: a meta-analysis. Clin Psychol Rev. 2007;27(3):318–26. doi: 10.1016/j.cpr.2006.11.001 17184887 10.1016/j.cpr.2006.11.001

[17] Mikulincer M, Shaver PR. Attachment in adulthood: structure, dynamics, and change. 2nd ed. New York: Guilford; 2016.

[18] Tol WA, Stavrou V, Greene MC, Mergenthaler C, Garcia-Moreno C, van Ommeren M. Mental health and psychosocial support interventions for survivors of sexual and gender-based violence during armed conflict: a systematic review. World Psychiatry. 2013;12(2):179–80. doi: 10.1002/wps.20054 23737431 10.1002/wps.20054 PMC3683274

[19] Rahman A, Hamdani SU, Riaz N, Bryant RA, Dawson K, Firaz M, et al Effectiveness of a psychological intervention using problem-solving and behavioral activation in adults with psychological distress in a conflict-affected area of Pakistan: a randomized clinical trial. JAMA. 2016;316(24):2609–17. doi: 10.1001/jama.2016.17165 27837602 10.1001/jama.2016.17165

[20] Sijbrandij M, Bryant RA, Schafer A, Dawson KS, Anjuri D, Ndogoni L, et al Problem Management Plus (PM+) in the treatment of common mental disorders in women affected by gender-based violence and urban adversity in Kenya; study protocol for a randomized controlled trial. Int J Ment Health Syst. 2016;10:44 doi: 10.1186/s13033-016-0075-5 27252778 10.1186/s13033-016-0075-5 PMC4888633

[21] Goldberg D, Williams PA. User’s guide to the General Health Questionnaire. Windsor: National Foundation for Educational Research; 1988.

[22] Ustun TB, Chatterji S, Kostanjsek N, Rehm J, Kennedy C, Epping-Jordan J, et al Developing the World Health Organization Disability Assessment Schedule 2.0. Bull World Health Organ. 2010;88(11):815–23. doi: 10.2471/BLT.09.067231 21076562 10.2471/BLT.09.067231 PMC2971503

[23] Minhas F, Mubbashar M. Validation of General Health Questionnaire (GHQ-12) in primary care settings of Pakistan. J Coll Physicians Surg Pak. 1996;6:133–6.

[24] World Health Organization. Measuring health and disability: manual for WHO Disability Assessment Schedule (WHODAS 2.0). Geneva: World Health Organization; 2010.

[25] Gray MJ, Litz BT, Hsu JL, Lombardo TW. Psychometric properties of the Life Events Checklist. Assess. 2004;11(4):330–41. 10.1177/1073191104269954 15486169

[26] World Health Organization. WHO multi-country study on women’s health and life experiences—final core questionnaire, version 10. Geneva: World Health Organization; 2003.

[27] Bernal G, Saez-Santiago E. Culturally centered psychological interventions. J Comm Psychol. 2006;34:121–32.

[28] Bolton P, Lee C, Haroz EE, Murray L, Dorsey S, Robinson C, et al A transdiagnostic community-based mental health treatment for comorbid disorders: development and outcomes of a randomized controlled trial among Burmese refugees in Thailand. PLoS Med. 2014;11(11):e1001757 doi: 10.1371/journal.pmed.1001757 25386945 10.1371/journal.pmed.1001757 PMC4227644

[29] Sikander S, Lazarus A, Bangash O, Fuhr DC, Weobong B, Krishna RN, et al The effectiveness and cost-effectiveness of the peer-delivered Thinking Healthy Programme for perinatal depression in Pakistan and India: the SHARE study protocol for randomised controlled trials. Trials. 2015;16:534 doi: 10.1186/s13063-015-1063-9 26604001 10.1186/s13063-015-1063-9 PMC4659202

[30] Kohrt BA, Jordans MJ, Rai S, Shrestha P, Luitel NP, Ramaiya MK, et al Therapist competence in global mental health: development of the ENhancing Assessment of Common Therapeutic factors (ENACT) rating scale. Behav Res Ther. 2015;69:11–21. doi: 10.1016/j.brat.2015.03.009 25847276 10.1016/j.brat.2015.03.009 PMC4686771

[31] Psychosocial Centre, International Federation of Red Cross and Red Crescent Societies, War Trauma Foundation, Danish Cancer Society, University of Innsbruck. Lay counselling: a trainer’s manual. Amsterdam: International Federation of Red Cross and Red Crescent Societies; 2013.

[32] World Health Organization. Psychological first aid: guide for field workers. Geneva: World Health Organization; 2011.

[33] Abubakar A, Fischer R. The factor structure of the 12-item General Health Questionnaire in a literate Kenyan population. Stress Health. 2012;28(3):248–54. doi: 10.1002/smi.1420 22282374 10.1002/smi.1420

[34] Getanda EM, Papadopoulos C, Evans H. The mental health, quality of life and life satisfaction of internally displaced persons living in Nakuru County, Kenya. BMC Public Health. 2015;15:755 doi: 10.1186/s12889-015-2085-7 26246147 10.1186/s12889-015-2085-7 PMC4527222

[35] Chepngeno-Langat G, Madise N, Evandrou M, Falkingham J. Gender differentials on the health consequences of care-giving to people with AIDS-related illness among older informal carers in two slums in Nairobi, Kenya. AIDS Care. 2011;23(12):1586–94. doi: 10.1080/09540121.2011.569698 22117125 10.1080/09540121.2011.569698 PMC3242068

[36] Weathers FW, Litz BT, Keane TM, Palmieri PA, Marx BP, Schnurr PP. The PTSD Checklist for DSM-5 (PCL-5). White River Junction (VT): National Center for PTSD; 2013.

[37] Ashworth M, Robinson S, Godfrey E, Shepherd M, Evans C, Seed P, et al Measuring mental health outcomes in primary care: the psychometric properties of a new patient-generated outcome measure, PSYCHLOPS (Psychological Outcome Profiles). Prim Care Ment Health. 2005;3:261–70.

[38] Whetten K, Ostermann J, Whetten R, O’Donnell K, Thielman N, Positive Outcomes for Orphans Research Team. More than the loss of a parent: potentially traumatic events among orphaned and abandoned children. J Traum Stress. 2011;24(2):174–82. 10.1002/jts.20625 PMC3610328 21442663

[39] Chisholm D, Knapp MR, Knudsen HC, Amaddeo F, Gaite L, van Wijngaarden B. Client Socio-Demographic and Service Receipt Inventory—European Version: development of an instrument for international research. EPSILON Study 5. European Psychiatric Services: Inputs Linked to Outcome Domains and Needs. Brit J Psychiatry Suppl. 2000;39:s28–33. 10.1192/bjp.177.39.s28 10945075

[40] Patel V, Weiss HA, Chowdhary N, Naik S, Pednekar S, Chatterjee S, et al Effectiveness of an intervention led by lay health counsellors for depressive and anxiety disorders in primary care in Goa, India (MANAS): a cluster randomised controlled trial. Lancet. 2010;376(9758):2086–95. doi: 10.1016/S0140-6736(10)61508-5 21159375 10.1016/S0140-6736(10)61508-5 PMC4964905

[41] Raudenbush SW. Comparing personal trajectories and drawing causal inferences from longitudinal data. Ann Rev Psychol. 2001;52:501–25. 11148315 10.1146/annurev.psych.52.1.501

[42] Singla DR, Kohrt B, Murray LK, Anand A, Chorpita BF, Patel V. Psychological treatments for the world: lessons from low- and middle-income countries. Ann Rev Clin Psychol. 2017;13:149–81. 28482687 10.1146/annurev-clinpsy-032816-045217 PMC5506549

[43] Patel V, Weobong B, Weiss HA, Anand A, Bhat B, Katti B, et al The Healthy Activity Program (HAP), a lay counsellor-delivered brief psychological treatment for severe depression, in primary care in India: a randomised controlled trial. Lancet. 2017;389:176–85. doi: 10.1016/S0140-6736(16)31589-6 27988143 10.1016/S0140-6736(16)31589-6 PMC5236064

[44] Weiss WM, Murray LK, Zangana GA, Mahmooth Z, Kaysen D, Dorsey S, et al Community-based mental health treatments for survivors of torture and militant attacks in Southern Iraq: a randomized control trial. BMC Psychiatry. 2015;15:249 doi: 10.1186/s12888-015-0622-7 26467303 10.1186/s12888-015-0622-7 PMC4605204

[45] Maselko J, Sikander S, Bhalotra S, Bangash O, Ganga N, Mukherjee S, et al Effect of an early perinatal depression intervention on long-term child development outcomes: follow-up of the Thinking Healthy Programme randomised controlled trial. Lancet Psychiatry. 2015;2(7):609–17. doi: 10.1016/S2215-0366(15)00109-1 26303558 10.1016/S2215-0366(15)00109-1

[46] Gold SM, Enck P, Hasselmann H, Friede T, Hegerl U, Mohr DC, et al Control conditions for randomised trials of behavioural interventions in psychiatry: a decision framework. Lancet Psychiatry. 2017 4 7 doi: 10.1016/S2215-0366(17)30153-0 10.1016/S2215-0366(17)30153-0 28396067

