# Supplementary material for: Effectiveness of a brief behavioural intervention on psychological distress among women with a history of gender-based violence in urban Kenya: A randomised clinical trial
Source: PLoS Med. 2017 Aug 15;14(8):e1002371. doi: 10.1371/journal.pmed.1002371 (PMC5557357; doi:10.1371/journal.pmed.1002371)
Supplement: S1 Text — (DOCX) [file pmed.1002371.s001.docx]

S1 TEXT: STUDY PROTOCOL­

Problem Management Plus (PM+) in the treatment of common mental disorders in women affected by adversity in urban Kenya

Study protocol for review by WHO Ethical Review Committee.

Version 4 (Amendment to previously accepted protocol)

March 12, 2015

**Note: Tracked changes in this document reflect proposed changes to the document after acceptance of Version 3 by the ERC Secretariat.**

*Contact person:*

Mark van Ommeren, PhD

World Health Organization (WHO)

Department of Mental Health and Substance Abuse

Email: [vanommerenm@who.int](mailto:vanommerenm@who.int)

Telephone: +41-22-791-3619

**Project summary**

*Background:* Common mental disorders (depression, anxiety disorders, posttraumatic stress disorder (PTSD)) are prevalent, especially after adversity. WHO has developed Problem Management Plus (PM+), a brief 5-session intervention, delivered by paraprofessionals (eg community health workers) for people affected by adversity.

*Objectives:* To test feasibility and acceptability of PM+ among women who have been directly or indirectly affected by urban violence in an exploratory randomized controlled trial (RCT; *Study Phase 1*); to perform a process evaluation after the exploratory RCT *(Phase 2)* evaluate effectiveness and cost-effectiveness of PM+ in a definitive RCT *(Phase 3);* and to perform a process evaluation after the definitive RCT (*Phase 4*).

*Design:* Qualitative studies (*Phases 2, 4*) and RCTs (*Phases 1, 3*).

*Methodology:* Informed by community consultations, the PM+ manual has already been translated and adapted to the local context by the counterpart agency. In Phase 1, the exploratory RCT will be in the catchment areas of 3 local health care facilities in Dagoretti Sub County, Nairobi. After informed consent, females with high psychological distress (General Health Questionnaire-12 (GHQ-12score>2) and functional impairment (WHO Disability Assessment Schedule 2.0 score>16) will be randomised to PM+ (n=20) or enhanced treatment as usual (TAU) (n=20). Post-treatment and 3-months post-treatment follow-up assessments also include psychological distress, functional disability, PTSD symptoms and perceived problems for which the person seeks help. Using a similar research design in Study Phase 3 a formal RCT will test effectiveness and cost-effectiveness of PM+ (173 in PM+, 173 in TAU). The process evaluations (Phase 2, 4) of RCTs) each involve 20 key informant interviews (KIs).

*Time* *frame*: 2 years.

*Expected* *outcomes*: Outcome will be a PM+ manual locally adapted for Kenya, and evidence concerning effectiveness and cost-effectiveness of PM+ in reducing common mental health problems among adversity-affected women in urban Kenya.

*Ethical considerations:* No minors will be involved. Confidentiality and anonymity will be protected. In Phases 1 and 3, the burden to adult participants consists of completing questionnaires and attending PM+ sessions. In Phases 2 and 4, the burden to adult participants and healthy volunteers consists of attending KI interviews. Risks to participants from the PM+ intervention are considered to be minimal. Since the proposed project may improve care for individuals with common mental disorders in Kenya, we believe that the major potential benefits outweigh the minor burden to participants.

**General information**

**Project title:**

Problem Management Plus (PM+) in the treatment of common mental disorders in women affected by adversity in urban Kenya.

**Name and address of the sponsor/funder:**

**World Vision Canada**

1 World Drive

Mississauga, Ontario

L5T 2Y4

Canada

**World Vision Australia**

1 Vision Drive, Burwood East,

Victoria,

Australia, 3151

**Grand Challenges Canada**

Sandra Rotman Centre

MaRS Centre, South Tower

101 College Street, Suite 406

Toronto, ON M5G 1L7

**Name and title of the investigators:**

Dr Mark van Ommeren

World Health Organization (WHO)

Department of Mental Health and Substance Abuse

Avenue Appia 20

1211 Geneva 27

Switzerland

Email: [vanommerenm@who.int](mailto:vanommerenm@who.int)

Telephone: +41-22-791-3619

*Responsibility:* Overall study coordination (Pakistan and Kenya)

Alison Schafer

World Vision Australia

1 Vision Drive

Burwood East, Victoria, 3155

Australia

Email: [alison.schafer@worldvision.com.au](mailto:alison.schafer@worldvision.com.au)

Telephone: +61 3 9287 2592

*Responsibility:* Project management

Jeannette Ulate, MD, MPH

World Vision Canada

1 World Drive

Mississauga, Ontario

L5T 2Y4 Canada

E-mail: [Jeannette_Ulate@worldvision.ca](mailto:Jeannette_Ulate@worldvision.ca)

*Responsibility*: Grant Manager/Technical Support

Lincoln Ndogoni

Consultant psychologist to World Vision Kenya

Address:

P.O. Box 17326-00100 GPO

Nairobi, Kenya

Email: [ndogoni@yahoo.com](mailto:ndogoni@yahoo.com)

Telephone: +254 733636513

*Responsibilities:* study preparation, training, supervision

Dorothy Anjuri

World Vision Kenya - Karen Office

P.O Box 50816 - 00200

Nairobi, Kenya

Email: [dorothy_anjuri@wvi.org](mailto:dorothy_anjuri@wvi.org)

Telephone: +254 20883652

*Responsibilities:* Site PI, study preparation, data collection (research site)

Prof. R. Bryant

University of New South Wales,

School of Psychology

Sydney, Australia

E-mail: [r.bryant@unsw.edu.au](mailto:r.bryant@unsw.edu.au)

*Responsibility:* Advice on intervention, training, data analysis, report

**International Advisors**

Dr Katie Dawson

University of New South Wales,

School of Psychology

Sydney, Australia

E-mail: [katie.dawson@unsw.edu.au](mailto:katie.dawson@unsw.edu.au)

*Responsibility:* Intervention development and training/supervision of health workers

Assistant Prof. Marit Sijbrandij

VU University Amsterdam,

Department of Psychology

E-mail: [e.m.sijbrandij@vu.nl](mailto:e.m.sijbrandij@vu.nl)

*Responsibility:* Advice on the study design and detailed research implementation protocols

**Address and telephone numbers of research site:**

World Vision Kenya

Riruta ADP

Phone: +254 724465893
Postal address: PO Box 50816-0200, Nairobi
Physical address: Equity Building, Kawangware, Nairobi

**Rationale and background information**

In poverty areas in low and middle income countries (LAMICs), common mental disorders (e.g., depression, anxiety disorders, and posttraumatic stress disorder (PTSD)) are very prevalent ((Kessler et al., 2008; Prince et al., 2007, Ormel et al., 2008) and are among the largest contributors to disability and functional impairment (Chisholm et al, 2007). Women living in urban poverty areas are especially vulnerable for common mental disorders, due to intimate partner violence or other types of violence, unwanted pregnancy (Dibaba et al., 2013) lack of resources, limited opportunities, concurrent physical health problems, low education, limited social supports, or other risk factors. These women tend to receive no or minimal mental health support (WHO, 2000).

In many low- and middle income countries, including urban slums, various nonspecific counselling programmes are practiced with unknown efficacy and safety. These may involve informal non-specific counselling without any manual describing which methods or procedures are used, and without any evidence for their efficacy. In addition, no or inadequate supervision of the persons delivering these interventions may be provided.

Therefore, effective treatments that target these common mental disorders, improve daily functioning and reduce health costs, should be developed and –if proven effective- widely disseminated across these areas. For interventions to be scalable, they should be of short duration and simple, so that they can be carried out by lay people in the community, such as community health workers (cf. Rahman, Malik, Sikander, Roberts, & Creed, 2008). Also, the interventions should address a range of outcomes, including general functioning and common mental and psychosocial health problems relevant to communities affected by adversity.

This project addresses these issues. We have developed an innovative, simplified psychological intervention *Problem Management Plus* *(PM+)* that has 4 core features. It is:

1. Brief (5-sessions),
2. Delivered by paraprofessionals,
3. Transdiagnostic, addressing depression, anxiety, PTSD, stress, and problems as defined by people themselves, and
4. Designed for people in low-income country communities affected by adversity (e.g. violence).

There is currently no programme that addresses all these features. PM+ consists of cognitive behavioural therapy (CBT) techniques (problem solving plus stress management, behavioural activation, facing fears, and accessing social support) that are empirically supported and formally recommended by the WHO (Dua et al., 2011; Tol, Barbui, & van Ommeren, 2013; WHO, 2010b, 2013).

**Study goals and objectives**

**Goal**:

To evaluate feasibility, acceptability and effectiveness of the locally adapted PM+ intervention among women in the Dagoretti Sub County , Nairobi, Kenya

**Specific objectives**

1. To test effectiveness and cost-effectiveness of PM+ in reducing common mental health problems, functional disability and health care costs in women affected by violence in Nairobi, Kenya*.*

2. To understand the perceptions of key stakeholders with regards to PM+ intervention.

**Study Design**

Exploratory single-blind randomised controlled trial (*Study Phase 1*), Key Informant (KI) interviews (*Phase 2*), definitive randomised controlled trial (*Phase 3*), KI interviews (*Phase 4*).

*Preparatory work (completed).* Before delivering a mental health intervention in a specific population it should be locally adapted (Bernal, 2006; Castro, Barrera, & Holleran Steiker, 2010). Through community consultations (cf. free listing interviews and key informant interviews) that have already been carried out by World Vision Kenya (without WHO involvement in ethnographic data collection or publication of results), priority problems and health concepts from the local perspective have been identified. World Vision Kenya prepared a community consultation report outlining key findings from their ethnographic study. It outlined the process of ethnographic free listing to determine major problems faced by this community, focus group discussions to explore these common problems and issues of violence as well as key informant interviews to better understand the referral and systematic processes existent in the community for supporting women affected by violence. The community consultations suggested that heavy alcohol use was a very serious and prevalent community concern, including due to its link with violence and domestic violence. Focus group discussions and key informant interviews ascertained that there are very few options for people affected by violence and that local community health workers felt a need to be trained in skills that equipped them to support women (in particular) who are living with and trying to manage such challenges.

The PM+ manual has been translated by expert Kiswahili linguists from University of Nairobi with a focus on keeping the language simple, the translation and adaptation have been reviewed in 2 workshops: one with experts on the intervention and the other with community health workers. Proposed adaptations have been recorded in the Bernal framework for documenting the adaptation of psychological interventions (Bernal, 2006; Chowdhary et al., 2013)). The PM+ translation in Kiswahili and adaption for the local context is considered ready for testing. Based on the information collected a module on brief interventions for hazardous alcohol use (based on existing WHO guidelines/manuals) will be added to PM+.

In *Study Phase 1,* we will conduct an exploratory randomised controlled trial (RCT) that will inform us about the feasibility and delivery of the intervention within a RCT; and that will identify issues around its training, supervision, randomization, recruitment, blinding, drop-out and outcomes measures. Our research strategy is informed by the *UK Medical Research Council framework for the development of complex interventions*, which recognizes iterations of: a) Intervention Development; b) Feasibility and Piloting; c) Evaluation; and d) Implementation (Craig et al., 2008). This key framework for development of interventions recommend feasibility and randomised pilot studies prior to large scale trials to address uncertainties such as problems of acceptability, compliance, delivery of the intervention, recruitment and retention (Craig et al., 2008).

In *Study Phase 3*, we will conduct a formal RCT, evaluating effectiveness and cost-effectiveness of PM+ in 346 study participants. See Figure 1 for an overview of the RCTs in phase 1 and 3.

Figure 1: Graphical outline of the design of the RCTs

Finally, in *study phases 2 and 4*, through KIs, we will evaluate barriers and facilitators among key stakeholders for large-scale implementation of PM+ through key informant interviews (participants, helpers, clinics heads and policy makers). Interviews will explore barriers and facilitators to treatment engagement and adherence. Phase 2 KIs results will help in carrying out the definitive trial. Phase 4 KI results will potentially inform scale up beyond the study area in Kenya.

**Methodology**

*Informed consent*

Before taking part in any interviews, oral and written information about the study and its purpose will be provided to respondents (see consent forms) in the local language. Immediately after completing informed consent, the interview will start. Respondents who decide to participate will be asked to complete a written consent form. For participants who are illiterate, witnessed oral consent and a thumb print in lieu of a signature will be sufficient. The witness will not be a member of the research team. Both of these alternatives to full written consent are routinely practiced in Kenya and are culturally acceptable.

*Participant reimbursement*

As a compensation for taking the time and effort of participating in the assessments, participants will receive reimbursements to the equivalent of 1000 Kenyan Shillings (10.92 USD) per assessment for the pre- and post-assessment and the 3-months follow-up assessments.

*Independent assessors and their supervisors*

Independent assessors are the people who screen and assess research participants. They will remain blind to treatment allocation during the RCTs. *Independent assessors will be recruited via a public recruitment process advertising the project and their roles. Selection criteria are the following:*

- *Form 4 level education plus a diploma in Counselling and/or social work or undergraduate students pursuing courses in psychology;*
- *Participated in previous surveys;*
- *Possess skills in data collection;*
- *Possess good interpersonal skills relevant for the interviews;*
- *Commitment and availability in completing the assignment (including for both Pilot and Definitive RCTs);*
- *Fluent in Kiswahili and English – ability to speak another language will be an added advantage.*

Twenty female independent assessors will be recruited. All 20 will be involved in the screening and in the pre and post assessments. A pool of candidates fitting the assessor criteria will be identified, with final selection of assessor made by the World Vision Field coordinator and World Vision Kenya site PI who has no personal knowledge of the candidates. Selection will be based upon fit to the assessor criteria, qualifications and skills and availability for the study timeline. During the recruitment process, assessors will be told interviewers about the potential stressful nature of the job. All assessor will receive a 5-day training on interview techniques, screening and assessment instruments and psychological first aid. A 1-day refresher training will be delivered by the investigators following the “just in time” training principle whereby training is delivered immediately prior to the conduct of interviews. During the project, the interviewers will also get support and supervision by the supervisor to handle possible difficulties they might experience

**Study Phase 1: Exploratory (Pilot) RCT to evaluate feasibility of administering the local adaptation of PM+ in a RCT setting and Study Phase 3: Definitive RCT to evaluate effectiveness and cost-effectiveness of the local adaptation of PM+ for use in Nairobi, Kenya**

Below we provide an overview of the design and methods of Study Phases 1 and 3. Note that the planned design and measures of Study Phase 1 and 3 are identical except for (a) the sample size and (b) the exploratory RCT does not involve a 3 months assessment follow-up. Study Phase 1 is an exploratory RCT that precedes the definitive RCT, and is intended to test the feasibility and acceptability of testing the PM+ intervention in a large RCT in Kenya, thereby testing the study procedures in the setting in which Study Phase 3 will be conducted.

*Overview*

In Study Phase 1, 40 females will be included (20 participants in each arm) and in Study Phase 3, a total of 346 females will be included (173 participants in each arm; see *Statistical power and sample size*). The study will be carried out in 3 local health care facilities that are part of the primary healthcare system of the Dagoretti Sub County Nairobi in Kenya. The study will be overseen by staff of World Vision Kenya and their Riruta Area Development Program (ADP)^[[1]](#footnote-2)^. Staffing at primary health care facilities changes, depending on staffing numbers and location, but at a minimum, they include a clinical officer, 3 nurses, a laboratory technician, a nursing assistant, and one counsellor, who is usually trained in simple counselling strategies, particularly for HIV/AIDS support. Each facility also employs a Community Health Extensive Worker (CHEW) who manages up to 100 volunteer Community Health Workers (CHWs) (though most health facilities have less than 50% CHW staffing capacity). Each CHW supports up to 100 households in their area. Overall, each health care facility in the research subcounty is usually catering for a mixed urban and semi-rural population of about 30,000.

*Participant inclusion and exclusion criteria* are: a) adult (18 years or older) female primary care attendees b) score above 2 on a screening questionnaire for common mental disorders (General Health Questionnaire-21; GHQ-12; Goldberg & Williams, 1988; Jenkins et al., 2013) (c) score above 16 on a screening questionnaire for functional impairments (WHO Disability Assessment Schedule 2.0; WHODAS; WHO, 2010a)). These instruments are described below. *Exclusion criteria are*: a) male gender; b) acute medical conditions; c) imminent suicide risk as defined in the mhGAP Intervention Guide (2010); or d) severe mental disorders or cognitive impairment (eg., severe intellectual disability, dementia, psychosis).

*Procedure*

1. Screening
   1. *Selection*. Participants will be included through community screening at the homes of the participants by the independent assessors. It is expected that 20% of the screened people will screen positive (Andrews et al 2009; Ustun & Sartorius, 1995) and that half of them would want to participate to research on PM+, so for the exploratory RCT 400 people will need to be screened and for the definitive trial 3460 people will need to be screened. Each CHW will have a list of the 100 households in the CHW’s catchment area. Using a random number table, a random sample will be taken from this list to decide which households will be approached. After selecting the households, independent assessors will identify and attempt to meet with the head of each household. The team will explain the purpose of the survey and ask if they may interview a random adult woman from the household. From the list of household members in each of the households surveyed, the assessor will randomly select 1 woman aged 18 years or older using a random number table. The selected person will be asked formal consent. Care will be taken that those screened for the exploratory RCT will not be screened for the definitive RCT.
   2. *Consent for screening.* The independent assessors will take informed consent before screening (see annex 1) As mentioned above, for participants who are illiterate, witnessed oral consent and a thumb print in lieu of a signature will be sufficient. The witness will not be a member of the research team. Participants will be free to decline to participate or withdraw at any time without affecting their routine care. If informed consent for screening is given, the same independent assessor will administer the screening instruments.
   3. *Confidentiality during screening.*

*The screeners who conduct the interviews to screen for eligibility in study phases 1 and 3, will be trained about the necessity of confidentiality of data privacy for individuals they are interviewing.*

*Interviews will be conducted face-to-face in a private space (e.g. in people’s homes or in a quiet area near their homes). If the woman has children older than 2 years old she has to take care of, she will be asked to arrange someone who could babysit for her during the interview. Women usually have freedom to speak about health matters in private.*

- 1. *Instruments.* Screening will be done using the instruments mentioned below.
  2. *Communication to people who screen negatively.* The independent assessors will refer individuals meeting any of the exclusion criteria (eg people with psychosis) to specialist support in the county according to their need. If participants are not selected because the score is below the cut-offs for the GHQ-12 or the WHODAS, they will be provided feedback on their test outcomes and will be explained why they are not eligible for the study. They will be referred to their CHW for potential follow-up. Participants with recently developed, acute protection risks will be offered psychological first aid by the assessor (WHO, War Trauma Foundation and World Vision International, 2011) and linked to available support services/resources. Participants with recent trauma will also be offered psychological first aid by the assessor. All independent assessors will be trained in the Kiswahili version of the WHO, War Trauma Foundation and World Vision International (2011) guide on psychological first aid). Participants with severe mental health disorders or organic disorders will be referred to the sub-county hospital. The assessor will contact the field coordinator who will ensure that an appointment is made, and with the person’s approval, the assessment results will be provided to the CHW in the area, who will be asked to help the person to attend the appointment at the hospital.
  3. *Ascent*. Detailed information on the next part of the study will be given to people who screen positive. Each person will be given at least 24 hours to think whether she wants to be part of the intervention study.

1. Informed consent for RCT and pre-assessment.
   1. *Appointment.* The assessor makes an appointment with the participant. This should be at least 1 day after the screening (but not longer than 3 days)
   2. *Informed consent.* At the appointment, the assessor will ask for informed consent for the trial (see annex 2). The written informed consent form (including who their assessment information is shared with) will be signed by the participant. As mentioned above, for participants who are illiterate, witnessed oral consent and a thumb print in lieu of a signature will be sufficient. The witness will not be a member of the research team.
   3. *Pre-assessment.* Next, pre-assessment is completed by administering the Life Events Checklist (LEC; (Gray, Litz, Hsu, & Lombardo, 2004)), the PTSD Checklist for DSM-5 (PCL-5, Weathers et al, 2013) and items derived from the Service Receipt Inventory (SRI; (Chisholm, Knapp, et al., 2000)).
2. Randomization
   1. Randomization will be carried out by an independent trial unit (at University of New South Wales) Randomization will be performed using computerised software on a 1:1 basis.
3. PM+ Intervention
   1. *Appointments.* CHW who will make contact with participants for treatment to begin. The first PM+ session will be scheduled within a few days and no longer than one week after the baseline assessment. This therapist will plan five consecutive meetings with the participant.
   2. *Additional instrument.* At the start of PM+ and at the beginning of every session the Psychological Outcome Profiles instrument (PSYCHLOPS; (Ashworth, 2004)) will be administered. This instrument, in contrast to above named instruments will be administered by the therapist *to assess and monitor* progress on problems for which the person seeks help.
4. Post intervention assessment
   1. *1-week follow-up.* The post-intervention assessment (WHODAS, GHQ-12, PCL-5) will be scheduled 7 weeks after the pre-intervention assessment (i.e., 1 week after the 5^th^ PM+ session).
   2. *3-month follow up.*  The follow-up assessment - with the same questionnaires plus the life events measure (LEC) and SRI items but without the treatment satisfaction questionnaire - will be scheduled at 3 months after the 5^th^ PM session.

All instruments will be administered by trained research staff blind to the allocation status of the participants. All independent assessors will receive a five day training in administering the instruments, in general interview techniques, and in responding to participant distress, including, as mentioned, psychological first aid.

*Statistical power and sample size*

Since Study Phase 1 is a small exploratory RCT with 40 participants (20 participants per group), that does not aim to detect statistically significant differences in effectiveness, no power calculations have been (or should be) carried out.

In Study Phase 3 a total number of 346 participants will to be included. Since we are not aware of intervention studies that have been carried out in this population, and we expect the population to be heterogeneous with respect to the types of common health disorders, we aimed for a relatively conservatively estimated 50% reduction in GHQ-12 symptom score in the PM+ group as compared to a 30% reduction in the control group at 3 months after the conclusion of the study. These estimates are in line with the observed effectiveness of an intervention led by lay health counsellors for depressive and anxiety disorders in a landmark study in primary care in India (Patel et al, 2010). This corresponds with an odds ratio of for the PM+ intervention of 2,3. Power calculations suggest a minimum sample size of 133 participants per group (power = 0.95, alpha = 0.05, two-sided). Taking into account an expected 30% attrition at 3 months follow-up, we aim to include a total number of 346 participants (173 in the PM+ group and 173 in the care-as-usual control group).

*Measures*

The measures for use in this study are in Annex 6.

The below matrix gives an overview of the measures used in the RCT in terms of when they will be assessed and what concepts they assess.

| *Concept* | Pre-assessment measures | Post-treatment assessment measures | 3-months post-treatment follow-up assessment measures |
| --- | --- | --- | --- |
| 1. functioning | WHODAS (**screener)** | WHODAS | WHODAS |
| 1. distress | GHQ-12 (**screener** and **primary outcome**) | GHQ-12 | GHQ-12 |
| 1. adverse life events | LEC |  | LEC |
| 1. violence against women | WHO-VAQ |  |  |
| 1. PTSD | PCL-5 | PCL-5 | PCL-5 |
| 1. Cost of care | SRI items |  | SRI items |

1. WHODAS: socio-demographic information and disability

Data on socio-demographic information (sex, age, education, marital status and work status) will be collected through questions A1-A5 of the WHO Disability Assessment Schedule 2.0 (WHODAS; [WHO, 2010a](#_ENREF_26)), which will be administered first. The WHODAS is a generic assessment instrument assessing health and disability. It is used across all diseases, including mental neurological and substance use disorders. It is simple to administer and applicable across cultures and can be used in all adult populations. WHODAS covers six domains (cognition, mobility, self-care, getting along, life activities, participation). It assesses difficulties people have due to their illness across these domains during the last 30 days. Difficulties are scored as none, mild, moderate, severe, or extreme. We will use the 12-item interviewer administered version. The WHODAS has been validated in Kenya (Chepngeno-Langat et al., 2011).

In analyses of Study Phase 3 data, the WHODAS will also be used to compute Quality Adjusted Life years, which is a measure of disease burden, for Study Phase 3 (Buttorff et al, 2012). Reference data for the female Kenyan population will be derived from the World Health Survey, in which Kenya was included (<http://apps.who.int/healthinfo/systems/surveydata/> index.php/catalog/80).

1. GHQ-12: distress

The primary outcome is level of psychological distress as measured by the General Health Questionnaire (GHQ-12; Goldberg & Williams, 1988; Jenkins et al., 2013) at 3 months follow-up (i.e., 3 months after the 5^th^ PM+ session). The GHQ-12 consists of 12 questions that are scored on a 4-point Likert scale ranging from 0 to 3..The total GHQ-12 score is obtained by summing up the scores of the individual items and ranges between 0-36 with higher scores representing higher levels of distress. When used as a screening tool, the GHQ-12 is usually scored bi-modally (i.e. - 0-0-1-1)., and ranges between 0-12. In a previous study in Kenya, a cut-off of higher than 2 has been reported to indicate clinical levels of distress (Jenkins et al., 2013).The GHQ has been widely used across low- and middle income countries, including Kenya (Jenkins et al., 2013).

1. LEC: exposure to potentially traumatic events (life events)

Previous stressor exposure will be assessed using the Life Events Checklist (Gray et al., 2004). This is a widely used list of 17 experienced or witnessed events, such as rape, serious injury, combat exposure, or the sudden death of a loved one. A Kiswahili version of this list is available (Whetten et al., 2011). At post-test the question phrasing will be adapted to capture life events that have occurred since commencing in the trial.

1. WHO Violence Against Women Instrument (WHO-VAW)

This study uses 5 key questions of the WHO Violence Against Women Instrument (WHO-VAW, WHO, 2003) as developed for use in the WHO Multi-Country Study on Women’s Health and Domestic Violence (WHO, 2005) and will adhere to the WHO ethical guidelines for the conduct of violence against women research, which stress confidentiality and privacy, and availability of referral for psychological and social help. The WHO Multi-Country Study on Women’s Health and Domestic Violence was conducted among 24 000 women in 10 countries, including Swahili-speaking Tanzania, and accordingly a Swahili version exists (WHO, 2005).

1. PCL-5: PTSD symptoms

Posttraumatic stress disorder (PTSD) symptoms will be measured using the PTSD Checklist for DSM-5 (PCL-5) (PCL-5; (Weathers et al, 2013), which is a 20-item checklist corresponding with the 20 DSM-5 PTSD symptoms. Items are rated on a 0-4 scale and add up to a total severity score of 80. The PCL-5 will be adapted to ask for symptoms in the last week (rather than month) to enhance sensitivity to change.

1. SRI: cost of care

The Service Receipt Inventory (SRI) was developed for the collection of data on service utilization and related characteristics of people with mental disorders, as the basis for calculating the costs of care for mental health cost-effectiveness research, including in low and middle income countries (Chisholm, Knapp, et al., 2000). Selected items from the SRI will be included, i.e., health seeking at the hospital, and from traditional healers.

*The PM+ intervention program:*

PM+ is a new, brief, WHO, psychological intervention program based on an evidence basis of established cognitive behavioural techniques with proven efficacy in low- and middle income countries (Dua et al, 2001, Tol et al , 2013). The full protocol was developed by WHO and University of New South Wales, Australia. The manual involves the following empirically supported elements: problem-solving, behavioural activation, facing unrealistic fears, accessing social support, and relaxation training. These elements have been recommended in recent WHO guidelines (Dua et al, 2011; Tol et al 2013).^.^

Next a brief outline of the five sessions is given.

Session 1:

- Introductions, assessment and consent form (30 minutes)
- Introduction to the program (20 minutes)
- Provide education: What happens after adversity? (20 minutes)
- Introduce ‘Managing Stress’ (relaxation techniques) (15 minutes)
- Set practice tasks and end the session (5 minutes)

Session 2:

- Give the client the during program assessment and use their responses to review the past week (5 minutes)
- Introduce ‘Managing Problems’ (problem solving) strategy and work through client’s main problem (40 minutes). In case alcohol use is a problem, *brief intervention* (motivational interviewing for alcohol) may be part of this (max 10 min).
- Introduce either ‘Get Going, Keep Doing’ or ‘Facing Fears’ (in vitro exposure exercises; depending on which is more appropriate due to client’s problems; 30 minutes)
- Practice ‘Managing Stress’ (10 minutes)
- Set practice tasks and end the session (5 minutes)

Session 3:

- During program assessment general review of the past week (5 minutes)
- ‘Managing Problems’ (25 minutes). In case alcohol use is a problem, *brief intervention* (motivational interviewing for alcohol) may be part of this (max 10 min).
- ‘Get Going Keep Doing’ or ‘Facing Fears’ (25 minutes; that is introduce the other strategy not completed last week and only if relevant to the client’s problems)
- Introduce ‘Strengthening Social Support’ (improving client’s social support network) (20 minutes)
- Practice ‘Managing Stress’ (10 minutes)
- Set practice tasks and end the session (5 minutes)

Session 4:

- During program assessment general review of the past week (5 minutes)
- ‘Managing Problems’ (25 minutes). In case alcohol use is a problem, *brief intervention* (motivational interviewing for alcohol) may be part of this (10 min).
- ‘Get Going Keep Doing’ or ‘Facing Fears’ (25 minutes)
- ‘Strengthening Social Support’ (20 minutes)
- Practice ‘Managing Stress’ (10 minutes)
- Set practice tasks and end the session (5 minutes)

Session 5:

- During program assessment general review of the past week (5 minutes)
- Discussion and education about how to stay well (30 minutes)
- Review PM+ strategies (30 minutes)
- Post-program assessment (20 minutes)
- Ending the session and program (5 minutes)

*Assessment as part of PM+:*

At the beginning of each PM+ session, participants will also be administered the Psychological Outcome Profiles instrument (PSYCHLOPS; [Ashworth, 2004](#_ENREF_2)) by their PM+ provider. PSYCHLOPS consists of four questions. It contains three domains: problems (2 questions), function (1 question) and wellbeing (1 question). Participants are asked to give free text responses to the problem and function domains. Responses are scored on an ordinal six-point scale producing a maximum score of 18 (6 points per domain). The peri (during)- and post-therapy versions of PSYCHLOPS consists of the same four questions but adds an overall valuation question (determining self-rated outcome ranging from “much better” to “much worse”). PSYCHLOPS has been validated in primary care populations across several countries (Czachowski, Seed, Schofield, & Ashworth, 2011; Heoinsson, Kristjansdottir, Olason, & Sigurosson, 2013).

*Enhanced Treatment-as-usual (TAU)*

Treatment-as-usual in these clinics in primary healthcare centres (PHCs) in Nairobi, Kenya to individuals with common mental disorders often consists of no treatment. For this study, participants in the TAU group will be referred to their primary care clinicians (usually nurses) for follow-up. These primary care nurses will have received the standard International Federation of Red Cross training in supportive counselling and psychological first aid (WHO, WTF, WVI, 2011) to enhance usual care.

If, during this treatment or during the study’s assessments participants in TAU arm show severe psychiatric disorders (eg psychosis) or problems (e.g., suicidality) that require immediate specialist treatment and follow-up, they will be referred to the subcounty hospital.

Of note, in Nairobi, each primary health care facility, and sub-country hospital, employs a Community Health Extension Worker (CHEW), who coordinates referrals from within the community and to ensure patients are followed-up by nurses and/or Community Health Workers (CHWs). The CHEWs from each health facility of the project is working with will be receiving a small additional allowance for their involvement in the research project. Their involvement will be to monitor that referred patients are actually seen when they are referred to the enhanced usual care or to the subcounty office. The CHEW will complete a basic form to monitor the date, time of the appointments and whether they occurred.

Throughout the trial, we will carefully keep track of the types and amount of support received through SRI items (see measures section above).

*PM+ providers and protocol adherence:*

PM+ providers will be female CHWs, who were recommended by the Ministry of Health, based on those that have shown high performance in their regular health work in the communities. After receiving the recommendation, CHWs will be selected based on individual interviews.

PM + providers will meet the following criteria:

- High school diploma (form 4 level of education or above);
- Reasonable proficiency in both Kiswahili and English language – written and verbal;
- 1-year training post-secondary school as CHWs or health related career;
- 2-years and above experience in community work;
- Already working as a CHW within a given geographical area.

PM+ providers will receive 2 weeks of training. In addition, one professional supervisors will be employed for each of the 3 health facility areas. Protocol adherence will be ensured by the supervisors and weekly group supervisions of the CHWs (Murray et al, 2011). Supervisors will then receive their own supervision and on-the-job training in supervision skills by the project lead consultant, who is a qualified counselling psychologist and available for any specific and/or urgent cases where either the CHWs themselves, the supervisors or any of the clients in the program need support. This will take place weekly as well.

To ensure adherence to the PM+ manual and ensure fidelity of interventions, 10% of all PM+ sessions will be attended by the supervisor, using a checklist to ensure basic elements of the PM+ intervention have been followed as required.

**Study Phase 2 and 4: Process evaluation of administering PM+ in Nairobi, Kenya**

The feasibility, and difficulties and successes in carrying out research and intervention activities will be explored through comprehensive process monitoring (see step 1 below) and semi-structured interviews with 20 KIs, including 5 CHWs (See step 2 below). Burden of completing the assessments and PM+ on the time and effort of participants, satisfaction with the intervention, and barriers and facilitators to adherence will be explored through semi-structured interviews with a sample of 5 participants (including participants that have dropped out). In addition, 5 decision-makers with responsibilities for developing or implementing health policy, including heads of the relevant clinics, and 5 PHC staff (clinical officers/nurses) will be interviewed to obtain their perceptions of the benefits and challenges of integrating PM+ into the CHWs routine service provision.

*Step 1. Process monitoring*

Process monitoring includes review of CHW records of sessions with clients; supervision records including intervention fidelity monitoring; and supervision of supervisors by intervention trainers. This data will be collected throughout the intervention delivery and reviewed as it is collected, leading to an iterative process of intervention monitoring informing intervention delivery. Confidential case notes and information will be handed to the research site for secure filing.

*Step 2: semi-structured interviews*

As mentioned above, additional individual semi-structured interviews will be conducted with 5 participants from each category of: intervention recipients; CHWs; clinical staff; and local stakeholders with a role in policy development or implementation. The aim of these interviews are to (a) make any necessary adjustments to the trial design (for KIs done after the exploratory RCT) and (b) explore the feasibility of wider implementation of the intervention in the Nairobi counties (for KIs done after the definitive RCT).Interviews will follow a semi-structured interview guide. A draft semi-structured interview guide has been developed (see Annex 5) with key questions that are identified for exploration, with additional prompt questions to fully explore each question in depth.

Interviews will follow the same process as outlined for Phase 1. Namely: informed consent will be obtained using the same informed consent procedure as in Phase 1 and 3 from all participants immediately prior to interviews (see KI consent form in annex 4); all interviews will be conducted by a pair of trained interviewers, one of whom will ask the questions and the other will record responses. No identifying information will be collected during the interview, with all data anonymised; and no recording devices will be used. Interviews will be conducted no longer than 4 weeks after the conduct of final outcome assessments, and are expected to last no longer than one hour. All key informant data will be analysed following thematic analysis

**Safety Considerations**

The World Vision Kenya team – which is very familiar with this subcounty – has confirmed, that CHWS and independent assessors can safely work in this community, including at people’s homes..

During the entire study, participants both in the PM+ or TAU arms will have access to enhanced usual care provided by trained primary care clinicians in the study area. If during the course of treatment, participants present with urgent psychiatric problems requiring referral to a specialist (e.g., a psychiatrist) or follow-up, this will be arranged with the CHW and health facility staff. Individuals will be referred to the to the Mbagathi sub county hospital.

Participants may experience benefits from the intervention that may be difficult for other family members to relate to. However, by carrying out community consultations before the project and in study phase 1 to ensure that PM+ is culturally acceptable, we expect that the intervention will not lead to behavior changes that are unacceptable within the local culture. In particular it should be noted that the intervention includes a focus on strengthening social supports in the participant’s lives. Participants with recently developed, acute protection risks will be offered psychological first aid by the PM+ provider (WHO, War Trauma Foundation and World Vision International, 2011) and linked to available support services/resources. So PM+ providers will have been trained to address this.

Information safety issues of the research staff and PM+ providers will be overseen by the Office of Corporate Security Team of World Vision Kenya, operating in their Nairobi HQ and providing security briefs to all World Vision Kenyan offices regarding security risks that may be imminent in certain areas. World Vision Kenya will not permit study members to engage in community work on days where insecurity is a risk. Therefore, all staff involved in the study have been advised that their wellbeing and safety come first and foremost, and that they are to end their work immediately if they become unsafe.

Safety of assessors will be ensured by the fact that assessors will be working in groups, dropped to particular areas each day and meeting up as specific times throughout their days. Thus, they will work in close proximity to each other and provide support and protection for each other.

The CHWs, who will provide the PM+ intervention, already visit specific households as a standard part of their work with health facilities in the area. A local security assessment has indicated that it will be safe for screeners, assessors and CHWs to work at the household level.

All assessors and PM+ providers will receive pre-paid phone credit by the project to facilitate constant communications with each other and with the local World Vision office.

At the end of the study (at 4-5 months after inclusion), participants in the TAU condition will also be offered the PM+ intervention, if still indicated. If after the project, participants continue to experience psychological problems they will be offered additional support through aforementioned referral hospital, see above).

All adverse reactions and serious adverse events (SAEs) reported spontaneously by the subject or observed by the investigators or other staff members will be recorded by the research team. We consider an event a SAE if it is an undesirable experience occurring to a subject during the study, whether or not considered related to the research procedure. Although it is unlikely that SAEs would occur given the nature of the intervention, all adverse events and SAEs will be reported to the local independent advisory board.

The local advisory board will consist of: an independent medical officer (chair), an independent counselling psychologist with community mental health experience, the site principle investigator, and the clinical supervisor. All serious adverse events (SAE) and adverse events (AE) will be reported by the local principle investigators or supervisor to this advisory board. The chair or a nominated person from the advisory board will review SAEs within 48 hours.

Terms of reference of the advisory board are . 1. to establish rules and procedures governing its own meetings, consistent with the research protocols and standard; 2. to review SAEs within 48 hours, and all (S)AEs once a month and determine whether an (S)AE is likely to be related or unrelated to the intervention. 3 as necessary, recommend appropriate action in respect of ongoing trial conduct (such as referral to specialized care or installing extra assessment points for monitoring participants), and 4. follow-up to know whether recommended actions have taken place

On the informed consent form, patient information is included to inform participants that the field coordinator, or another clinician other than their therapist are available to them if they are upset by this study.

The principal investigator will inform the subjects and the WHO ERC if anything occurs, on the basis of which it appears that the disadvantages of participation may be significantly greater than was foreseen in the research proposal.

**Follow-Up**

The exploratory RCT and definitive RCT differ in follow-up assessments. While in the exploratory RCT, the final post intervention assessment by independent assessors is 1 week after the completion of PM+, in the definitive RCT the final outcome measures will be taken at 3 months after the final PM+ session. As mentioned, 5 participants will also be invited to participate in semi-structured interviews. These interviews will be conducted within 4 weeks of the final outcome assessment (i.e within 4 months of the intervention). All participants will be told that they may contact their local primary health care facility in case they need future support. Names and contact details of the field coordinator and local site PI will be provided on the consent form of which all participants receive a copy.

Depending on the adverse event, follow up may require additional tests or medical procedures as indicated, and/or referral to the general physician or a medical specialist. All adverse events will be followed until specialist care (including referrals, additional tests or medical procedures) is in place for the client, or until a stable situation has been reached.

**Data Management and Statistical Analysis**

The data, as collected by the interviewers (Study Phases 2 and 4) and independent assessors and PM+ providers (Study Phases 1 and 3) will be safely stored at the World Vision office of the field coordinator at Rituta ADP

Quantitative data will be coded and the identifying key (a list connecting names to numbers) will be kept in a separate, secure locked location in the field coordinator’s office. The data will be entered into a data-analytic computer program (e.g., SPSS), without the identifying key. Data will only be available to the members of the project group. No attributable data will be used in publications. The investigators will analyze the data, and both positive and negative trial results will be disclosed. Results will be submitted for publication to peer-reviewed scientific journals.

The qualitative research will follow thematic analyses as described above.

For the RCT’s, both intention-to-treat analysis and completers’ analyses will be carried out. To measure comparisons at baseline between the two treatment groups t-tests will be conducted for continuous variables and chi-squared test for categorical ones. Repeated measurement analysis will be carried out to assess differential change over time in mean GHQ-12 scores between groups. We will add the following covariates at baseline to examine subgroup effects: education, employment, and severity of symptoms. All analyses will be carried out in SPSS, with p-levels of <.05 indicating statistical significance.

For the economic evaluation, we follow the approach used before in a similar trial (Buttorff et al, 2012). The number of days with reduced working hours because of poor health will be calculated from the WHODAS-12. Participants’ self-reported their health-care utilization, medication use and out-of-pocket payments will be measured with the items derived from the SRI at post treatment and 3 months follow-up.

For the costs analysis, two types of costs measured at post-treatment and at the 3-months follow-up will be estimated based on SRI scores: health-system costs (i.e., costs related to the intervention itself and associated medications and/ or clinical investigations) and the "time costs" for the subjects and their families (i.e. time spent travelling to, waiting for or receiving care, plus the wages from any days of work lost). The human resource use associated with the community health workers employed in the intervention will be estimated by multiplying the time a participant in PM+ has contact with a CHW by the per-hour cost of the CHW.

Costs, scores and days-of-work data from each follow-up will be summed to give totals for the 3 months post-treatment. Differences between PM+ and TAU, will be assessed using generalized linear models. Cost–effectiveness acceptability curves showing the probability that an intervention remains relatively cost–effective at increasing monetary values, will be developed.

**Quality Assurance**

Participants in the RCTs may directly benefit from their participation in the PM+ intervention, which is based on cognitive behavioural therapy (CBT) techniques (problem solving, stress management, behavioural activation, facing fears, and accessing social support) that are empirically supported and recommended by the WHO for use in low and middle income countries (Dua et al., 2011; Tol et al., 2013; WHO, 2010b). Their participation will furthermore inform local application of PM+ manual, and thus improve knowledge about delivering mental health interventions in the study area. Since PM+ is non-pharmacological and there is a broad evidence base for its use, it is unlikely that adverse effects due to the intervention will occur in Study Phases 1 and 3. Therefore, we believe that it is not necessary to install a Data Monitoring Safety Board for this study.

**Expected Outcomes of the Study**

We expect that the current study will advance knowledge about the feasibility and acceptability of implementing PM+ among women in adversity-affected communities in LAMIC. PM+ is a scalable intervention that mobilizes resources already available in settings with a lack of mental health care such as the Dagoretti Sub County, Nairobi, Kenya. PM+ is innovative in that it is simplified, low-intensity and adaptable intervention that can be delivered by supervised community health workers. Thus, it has the potential to provide the much-needed template for context-specific interventions that may not only be used in similar areas in Kenya, but rolled out to other areas for further adaptation and testing in diverse settings.

**Dissemination of Results and Publication Policy**

Results of the study will be shared with provincial and national government, county, and national health authorities have already been informed about the study, with the Kenya MoH recognised as a formal partner for the research project. In addition, all research participants will be informed about the results of the project by their community health workers.

Dissemination will also be done through World Vision to international networks in health and mental health groups to pave the way for future government and NGO partners to use these materials in other contexts.*

Results of the project will also be disseminated in English scientific peer-reviewed journals as well as in Kiswahili to key stakeholders (eg. heads of relevant health services, participants in previous community engagement meetings).

With respect to dissemination in the scientific media, we expect at least the following outputs: 1) a paper describing the qualitative assessment and the local cultural adaptation of PM+ 2) a paper describing the results of the randomised controlled trial in an international peer-reviewed journal.

An exploratory RCT to test feasibility and acceptability of PM+ in Pakistan is being carried out at the moment. Approval for this study, which is very similar to the current study, has been obtained from WHO’s Ethical Review Committee (Protocol ID RPC627). A grant proposal for a larger RCT in Pakistan has recently been submitted. In case the RCT’s show definite effectiveness of PM+ in reducing common mental health problems in Kenya, Pakistan and other low and middle income countries, it will be made widely available as part of WHO’s flagship mhGAP programme (WHO, 2010b). In addition, dissemination workshops for local health workers and policy makers would be organised.

**Duration of the Project**

|  | |
| --- | --- |
| **Study Phase 1: Exploratory randomised controlled trial** | |
| **Activities** | **Month** |
| Selection and training of CHWs (PM+ providers) and their supervisors and independent assessors | February 2014 |
| Participants are identified | March 2014 |
| Pre-intervention data are collected | April 2014 |
| CHWs (PM+ providers) implement PM+ | May-July 2014 |
| Post intervention data is collected from service users and control group | August 2014 |
|  |  |
| Control group participants who have not shown improvements are referred for PM+ | August 2014 |
| Data entry | August-Sept 2014 |
| Analysis and report of exploratory RCT results | October-Nov 2014 |
| **Study Phase 2: Process evaluation of exploratory RCT** | |
| **Activities** |  |
| Process monitoring | September 2014 |
| Qualitative interviews | September 2014 |
| **Study Phase 3: Definitive randomised controlled trial** | |
| **Activities** | **Month** |
| Pre-intervention data are collected | December 2014 |
| CHWs (PM+ providers) implement PM+ | Jan-May 2015 |
| Post intervention data is collected from service users and control group | June 2015 |
| month follow up data is collected | August 2015 |
| Control group participants who have not shown improvements are referred for PM+ | July 2015 |
| Data entry | Dec 2014 – August 2015 |
| Analysis and preparation of manuscript describing effectiveness and cost-effectiveness of PM+ in Nairobi, Kenya | August-Sept 2015 |
|  |  |
| **Study Phase 4: Process evaluation of definitive RCT** | |
| **Activities** |  |
| Process monitoring | August 2015 |
| Qualitative interviews | August 2015 |

**Problems Anticipated**

We foresee no problems in completing the study phases 1, 2, 3 and 4 within the proposed time frame. Since the project group members are locally-based and are experienced in managing projects in this community.

**Project Management**

Dr Mark van Ommeren, Scientist at WHO, is focal point for WHO for mental health in emergencies. He is experienced in leading complex policy and research projects to timely completion. He will be responsible for the overall study coordination in Kenya and Pakistan (Previously approved project, see Protocol ID RPC627).

Dr Jeannette Ulate, World Vision Canada Health Technical Specialist/Project Design and Development. She is a Senior Manager with more than 20 years of international experience; she will be the Grant Manager of the project in Kenya and the communication focal point for Grant Challenges Canada.

Alison Schafer of World Vision Australia – who led the drafting of the proposal for this project - will oversee the project management. She is an experienced project manager and familiar with Kenya context.

Dorothy Anjuri from Kenya is the local site PI. She has experience in project management of large World Vision projects in Kenya and selected other countries in Africa. She will oversee and support the work by the Field coordinator .

Lincoln Ndogoni is consultant psychologist to World Vision Kenya and he will oversee the training of the CHWS. He has done the same for World Vision in a JAMA published trial on interpersonal therapy (Bolton et al, 2004).

Richard Bryant and Marit Sijbrandij– who together have the experience of having led 20 RCTs - are the methodologists for this trial and will advise on all aspects related to the trial and intervention development.

**Ethics**

The project protocol will be reviewed by the Great Lakes University of Kenya, Nairobi and the WHO Ethics Review Committee.

No minors will be involved in this study. All participants will be adults older than 18 years.

Full information on the study will be provided in local, lay language before consent (written or oral as described above) will be obtained from each participant. This information will be read out to participants who are illiterate in the presence of a witness not affiliated with the study. We will be careful to ensure that potential participants clearly understand the implications of their involvement and that (a) they can withdraw their consent at any time without giving an explanation; (b) refusal or withdrawal of consent would have no impact upon any type of support they receive. The informed consent procedure is described in this protocol, and follows recommendations by WHO’s Ethical Review Committee (<http://www.who.int/rpc/research_ethics/Process_seeking_IF_printing.pdf>). This procedure includes: 1) oral and written information to consider participation; 2) a variant for illiterate participants, who may give consent through both a signature of a literate witness (not a member of the research team) and a thumb print.

People with recently developed, acute protection risks or recent trauma (i.e., in the past month) will be offered psychological first aid by the assessor (WHO, War Trauma Foundation and World Vision International, 2011) and linked to available support services/resources. Participants with recent trauma will also be offered psychological first aid by the assessor. All independent assessors will be trained in the Kiswahili version of the WHO, War Trauma Foundation and World Vision International (2011) guide on psychological first aid.

This study uses 5 key questions of the WHO Violence Against Women Instrument (WHO-VAW, WHO, 2003) as developed for use in the WHO Multi-Country Study on Women’s Health and Domestic Violence (WHO, 2005) and will adhere to the WHO guidelines for the conduct of violence against women research, which stress confidentiality and privacy, and availability of referral for psychological and social help. Before asking these 5 questions, women will be reminded that they are free to skip any questions. To ensure confidentiality, the interviewers are trained that of they are interrupted they will either terminate the interview, or to stop asking about violence and to move on to another, less sensitive topic until privacy can be ensured.

Another concern may involve the fact that participants in PM+ will receive treatment, whereas participants in TAU will not receive PM+. However, PM+ is a new intervention in this population, and although the efficacy of its main cognitive behavioral therapy components has been established in other populations (Dua et al., 2011; Tol et al., 2013; WHO, 2010b), a RCT is the necessary step for establishing effectiveness. In addition, participants in TAU will not be withheld care, but rather will have access to enhanced TAU. If, during the study participants in either arm of the study show severe psychiatric disorders or problems (e.g., suicidality) that require immediate treatment and follow-up, they will be referred to the relevant referral hospital.

The study is done in a low-income context, in which many individuals are exposed to multiple stressors and traumatic events. To test the utility of the PM+ protocol in real-world settings, it must be tested in situations in which it is intended to be implemented where there are ongoing stressors, uncertainty, adverse life events, etc. The current study is a necessary next step to test whether an intervention such as PM+ may be feasible and acceptable in such context with ongoing stressors and uncertainty. PM+ is based on evidence-based psychological techniques, that have been proven effective in more stable settings (WHO, 2013). Evaluating the intervention for delivery via non-specialist CHWs and testing their training and supervision procedures has to be carried out in real-life settings to provide meaningful information for implementation and scale-up.

**Informed Consent Forms**

Informed consent forms have been uploaded.

**Budget**

#### The budget has been uploaded. Note that while the trial is largely supported by Grand Challenges Canada, that World Vision Canada supports WHO’s involvement at a budget of USD 70,000

#### Other support for the project

#### None

#### Collaboration with other scientists or research institutions

#### The scientists collaborating in this project are all listed in the General Information section of this document.

#### The study involves the following institutions (see section on project management):

1. World Vision in collaboration with Ministry of Health Kenya
2. WHO.

#### Ethical clearance for the work by World Vision in Kenya will be locally obtained.

The project has 2 advisors:

1. Prof Richard Bryant (University of New South Wales, Australia) is a world-leading researcher on adversity and has conducted trials in humanitarian settings in Australia, Indonesia and Thailand. He will be responsible for advising on research design and the psychological intervention.
2. Dr Marit Sijbrandij (VU University Amsterdam, the Netherlands), has conducted trials on populations recently exposed to adversities, including a key trial showing the ineffectiveness of debriefing. She will be responsible for advising on research design and detailed research protocols

#### All scientists, including the 2 advisors, have completed declaration of interest forms, and none have declared an interest.

#### Links to other projects

#### There is a link to the previously approved research project (Protocol ID RPC627) of WHO in collaboration with 3 Pakistan-based institutions (profs. A. Rahman, Saeed Farooq and Fareed Minhas) and UNSW to test the PM+ manual in Peshawar, Pakistan in an exploratory RCT

#### Other research activities of the investigators

#### Other research activities by the investigators are summarised in the attached cv’s. In addition, the WHO research coordinator is collaborating on a Cochrane Review on mental health and psychosocial support interventions in humanitarian settings

#### Financing and Insurance

We consider the study as low-risk. Therefore, insurance is not deemed necessary.

**References**

Ahmer, S., Faruqui, R. A., & Aijaz, A. (2007). Psychiatric rating scales in Urdu: a systematic review. *BMC Psychiatry, 7*, 59. doi: 10.1186/1471-244X-7-59

Ashworth, M.; Shepherd, M.; Christey, J.; Matthews, V.; Wright, K.; Parmentier, H.; Robisnon, S.; & Godfrey, E. (2004). A client-generated psychometric instrument: The development of ‘PSYCHLOPS’. *Counselling and Psychotherapy Research: Linking research with practice, 4*(2), 27-31.

Andrews G, Kemp A, Sunderland M, Von Korff M, Ustun TB. Normative data for the 12 item WHO Disability Assessment Schedule 2.0. *PLoS One.* 2009 Dec 17;4(12):e8343.

Bass, J.K., Annan, J., McIvor Murray, S., Kaysen, D., Griffiths, S., Cetinoglu, T., Wachter, K., Murray, L.K., Bolton, P.A. (2013). Controlled trial of psychotherapy for Congolese survivors of sexual violence. New England Journal of Medicine, 6;368(23), 2182-9

Bernal, G.; Saez-Santiago, E.;. (2006). Culturally centered psychological interventions. *Journal of Community Psychology, 34*(2), 121-132.

Betancourt, T. S., Speelman, L., Onyango, G., & Bolton, P. (2009). A qualitative study of mental health problems among children displaced by war in northern Uganda. *Transcult Psychiatry, 46*(2), 238-256. doi: 10.1177/1363461509105815

Bolton, P., Surkan, P. J., Gray, A. E., & Desmousseaux, M. (2012). The mental health and psychosocial effects of organized violence: a qualitative study in northern Haiti. *Transcult Psychiatry, 49*(3-4), 590-612. doi: 10.1177/1363461511433945

Bolton, P.; Tol, W.; and Bass, J. (2009) Introduction to special issue: Combining qualitative and quantitative research methods to support psychosocial and mental health programmes in complex emergencies; Intervention, Vol. 7, No. 3, pp. 181 - 186

Buttorff, C., Hock, R. S., Weiss, H. A., Naik, S., Araya, R., Kirkwood, B. R., . . . Patel, V. (2012). Economic evaluation of a task-shifting intervention for common mental disorders in India. Bull World Health Organ, 90(11), 813-821. doi: 10.2471/BLT.12.104133

Castro, F. G., Barrera, M., Jr., & Holleran Steiker, L. K. (2010). Issues and challenges in the design of culturally adapted evidence-based interventions. Annu Rev Clin Psychol, 6, 213-239. doi: 10.1146/annurev-clinpsy-033109-132032

Checchi, F.; Gayer, M; Grais, R; Mills, E;. (2007). Public Health in crisis affected populations. HPN Network Paper, 67.

Chepngeno-Langat, G., Madise, N,, Evandrou, M., Falkingham, J. (2011). Gender differentials on the health consequences of care-giving to people with AIDS-related illness among older informal carers in two slums in Nairobi, Kenya. [AIDS Care,](http://www.tandfonline.com/toc/caic20/23/12) 23 (12).

Chisholm,  D.,  Lund,  C.,  Saxena,  S. (2007).  Cost of scaling up mental healthcare in low- and middle- income countries.  British Journal of Psychiatry, 191,528–535.

Chisholm, D., Knapp, M. R., Knudsen, H. C., Amaddeo, F., Gaite, L., & van Wijngaarden, B. (2000). Client Socio-Demographic and Service Receipt Inventory--European Version: development of an instrument for international research. EPSILON Study 5. European Psychiatric Services: Inputs Linked to Outcome Domains and Needs. Br J Psychiatry Suppl(39), s28-33.

Chisholm, D., Sekar, K., Kumar, K. K., Saeed, K., James, S., Mubbashar, M., & Murthy, R. S. (2000). Integration of mental health care into primary care. Demonstration cost-outcome study in India and Pakistan. *Br J Psychiatry, 176*, 581-588.

Chowdhary, N., Jotheeswaran, A. T., Nadkarni, A., Hollon, S. D., King, M., Jordans, M. J., . . . Patel, V. (2013). The methods and outcomes of cultural adaptations of psychological treatments for depressive disorders: a systematic review. *Psychol Med*, 1-16. doi: 10.1017/S0033291713001785

Craig, P., Dieppe, P., Macintyre, S., Michie, S., Nazareth, I., Petticrew, M., & Medical Research Council, Guidance. (2008). Developing and evaluating complex interventions: the new Medical Research Council guidance. *BMJ, 337*, a1655. doi: 10.1136/bmj.a1655

Czachowski, S., Seed, P., Schofield, P., & Ashworth, M. (2011). Measuring Psychological Change during Cognitive Behaviour Therapy in Primary Care: A Polish Study Using 'PSYCHLOPS' (Psychological Outcome Profiles). *Plos One, 6*(12). doi: ARTN e27378

Dhadphale M, Ellison RH, Griffin L. (1983). The frequency of psychiatric disorders among patients attending semi-urban and rural general out-patient clinics in Kenya. *Br J Psychiatry, 142,* 379-83.

Dibaba, Y., Fantahun, M., Hindin, M.J. (2013). The association of unwanted pregnancy and social support with depressive symptoms in pregnancy: evidence from rural Southwestern Ethiopia. BMC Pregnancy Childbirth, 24 (13), 135.

Dua, T., Barbui, C., Clark, N., Fleischmann, A., Poznyak, V., van Ommeren, M., . . . Saxena, S. (2011). Evidence-based guidelines for mental, neurological, and substance use disorders in low- and middle-income countries: summary of WHO recommendations. *PLoS Med, 8*(11), e1001122. doi: 10.1371/journal.pmed.1001122

Goldberg D, Williams P: A user’s guide to the General Health Questionnaire.

Windsor, UK: NFER- Nelson; 1988.

Gray, M. J., Litz, B. T., Hsu, J. L., & Lombardo, T. W. (2004). Psychometric properties of the life events checklist. *Assessment, 11*(4), 330-341. doi: 10.1177/1073191104269954

Heoinsson, H., Kristjansdottir, H., Olason, D. P., & Sigurosson, J. F. (2013). A Validation and Replication Study of the Patient-Generated Measure PSYCHLOPS on an Icelandic Clinical Population. *European Journal of Psychological Assessment, 29*(2), 89-95. doi: Doi 10.1027/1015-5759/A000136

Husain, N., Chaudhry, N., Fatima, B., Husain, M., Amin, R., Chaudhry, I. B., . . . Creed, F. (2010). Antidepressants and Group Psychosocial Treatment for Depression: An Rct from a Low Income Country. *European Psychiatry, 25*.

Jenkins R, Njenga F, Okonji M, Kigamwa P, Baraza M, Ayuyo J, Singleton N, McManus S, & Kiima D. (2012). Prevalence of common mental disorders in a rural district of Kenya, and socio-demographic risk factors. Int. J. Environ. Res. Public Health, 9, 1810-1819.

Jenkins R, Othieno C, Okeyo S, Kaseje D, Aruwa J, Oyugi H, Bassett P, Kauye F. (2013). Short structured general mental health in service training programme in Kenya improves patient health and social outcomes but not detection of mental health problems - a pragmatic cluster randomised controlled trial. Int J Ment Health Syst. 7(1):25.

Kessler, R.C., & Üstün, T. B. (Eds.). (*2008).* The WHO World Mental Health Surveys: global perspectives on the epidemiology of mental disorders*.* New York: Cambridge University Press, 1-580.

Khalily, M. T. (2011). Mental health problems in Pakistani society as a consequence of violence and trauma: a case for better integration of care. *Int J Integr Care, 11*, e128.

Khalily, M.T., Gul, S., Mushtaq, R., Jahangir, S.F. (2012). To examine delayed PTSD symptomatology over time among trauma survivors in Pakistan. *The Online Journal of Counselling and Education, 1*(1), 1-11.

Lewis, G., Pelosi, A. J., Araya, R., & Dunn, G. (1992). Measuring psychiatric disorder in the community: a standardized assessment for use by lay interviewers. *Psychol Med, 22*(2), 465-486.

Murray, L. K., Singh, N. S., Surkan, P. J., Semrau, K., Bass, J., & Bolton, P. (2012). A qualitative study of georgian youth who are on the street or institutionalized. *Int J Pediatr*, 2012, 921604. doi: 10.1155/2012/921604

Murray, L., Dorsey, S., Bolton, P., Jordans, M., Rahman, A., Bass, J. & Verdeli, H. (2011) Building capacity in mental health interventions in low resource countries: an apprenticeship model for training local providers; *International Journal of mental health* systems; 5, 30 - 42

Ormel,  J.M.,  Petukhova,  S.;  Chatterji,  S.  et al (2008). Disability and treatment of specific mental and physical disorders across the world.  British Journal of Psychiatry 192, 368–375, 2008

Patel V, Weiss HA, Chowdhary N, Naik S, Pednekar S, Chatterjee S, De Silva MJ, Bhat B, Araya R, King M, Simon G, Verdeli H, Kirkwood BR. Effectiveness of an intervention led by lay health counsellors for depressive and anxiety disorders in primary care in Goa, India (MANAS): a cluster randomised controlled trial. Lancet. 2010 Dec 18;376(9758):2086-95. doi: 10.1016/S0140-6736(10)61508-5.

Prince,  M.,  Patel,  V.  Saxena,  S., [Maj](http://www.thelancet.com/search/results?fieldName=Authors&searchTerm=Mario+Maj), M., [Maselko](http://www.thelancet.com/search/results?fieldName=Authors&searchTerm=Joanna+Maselko), J., Phillips, M.R., Rahman, A. (2007). No health without mental health.  Lancet, 370, 859–77.

Rahman, A., Malik, A., Sikander, S., Roberts, C., & Creed, F. (2008). Cognitive behaviour therapy-based intervention by community health workers for mothers with depression and their infants in rural Pakistan: a cluster-randomised controlled trial. Lancet, 372(9642), 902-909.

Saunders JB, Aasland OG, Babor TF, de la Fuente JR, Grant M. Development of the Alcohol Use Disorders Identification Test (AUDIT): WHO Collaborative Project on Early Detection of Persons with Harmful Alcohol Consumption--II. Addiction. 1993 Jun;88(6):791-804.

Sebit MB. (1996). Prevalence of psychiatric disorders in general practice in Nairobi. East Afr Med J. 73(10):631-3.

Tol, W. A., Barbui, C., Galappatti, A., Silove, D., Betancourt, T. S., Souza, R., . . . van Ommeren, M. (2011). Mental health and psychosocial support in humanitarian settings: linking practice and research. *Lancet, 378*(9802), 1581-1591. doi: 10.1016/S0140-6736(11)61094-5

Tol, W. A., Barbui, C., & van Ommeren, M. (2013). Management of acute stress, PTSD, and bereavement: WHO recommendations. *JAMA, 310*(5), 477-478. doi: 10.1001/jama.2013.166723

Ustun, T. B., Sartorius N. *Mental Illness in General Health Care: An International Study*. John Wiley, 1995.

Weathers, F.W., Litz, B.T., Keane, T.M., Palmieri, P. A., Marx, B. P., & Schnurr, P.P. (2013). The PTSD Checklist for DSM-5 (PCL-5).. Boston, MA.

Whetten K, Ostermann J, Whetten R, O'Donnell K, Thielman N. (2011). [More than the loss of a parent: potentially traumatic events among orphaned and abandoned children.](http://www.ncbi.nlm.nih.gov/pubmed/21442663) Positive Outcomes for Orphans Research Team*. J Trauma Stress,* 24(2), 174-82.

Whiteford, H. A., Degenhardt, L., Rehm, J., Baxter, A. J., Ferrari, A. J., Erskine, H. E., . . . Vos, T. (2013). Global burden of disease attributable to mental and substance use disorders: findings from the Global Burden of Disease Study 2010. *Lancet*. doi: 10.1016/S0140-6736(13)61611-6

WHO. (1994a). The ICD-10 Classification of Mental and Behavioural Disorders:Diagnostic Criteria for Research. Geneva, Switzerland.

WHO. (2000). Women’s mental health: an evidence based review. WHO: Geneva.

WHO (2003). WHO Multi-Country Study on Women’s Health and Life Experiences - Final Core Questionnaire, version 10. WHO: Geneva

WHO (2005). WHO multi-country study on women’s health and domestic violence against women: summary report of initial results on prevalence, health outcomes and women’s responses. WHO: Geneva.

WHO. (2010a). Measuring health and disability; Manual for WHO Disability Assessment Schedule WHODAS 2.0. Geneva.

WHO. (2010b). MhGAP intervention guide for mental, neurological and substance use disorders in non-specialized health settings. Geneva: WHO.

WHO. (2013). Guidelines for the management of conditions specifically related to stress. Geneva: World Health Organization.

WHO/UNHCR. (2013). Assessment and Management of Conditions Specifically Related to Stress: mhGAP Intervention Guide Module (version 1.0). Geneva, Switzerland.

WHO, War Trauma Foundation and World Vision International (2011). Psychological first aid: Guide for field workers. WHO: Geneva.

1. ADP is a World Vision Term that stands for “Area Development Program”. It is best described as a geographic area where World Vision implements long-term community development programs, beginning with community consultations and needs assessments and strategic work with local leaders and community members on facilitating their realization of those needs. ADPs usually run for 10-15 years and support directly or indirectly up to (give or take) 30,000 men, women and children [↑](#footnote-ref-2)
