# Supplementary material for: Effectiveness of a brief behavioural intervention on psychological distress among women with a history of gender-based violence in urban Kenya: A randomised clinical trial
Source: PLoS Med. 2017 Aug 15;14(8):e1002371. doi: 10.1371/journal.pmed.1002371 (PMC5557357; doi:10.1371/journal.pmed.1002371)
Supplement: S3 Text — (DOCX) [file pmed.1002371.s003.docx]

S3 TEXT: SUPPLEMENTARY ANALYSES

*Service Utilization*

Regarding service utilization, there were no differences between PM+ and EUC conditions at the 3-month follow-up on whether there had been a hospital admission [2.56% vs 3.07%, OR 1.13 [95% CI, 0.52 – 2.45], *P* = .75), or the number of out-patient consultations (*M* = 1.62, *SD* = 2.56 vs *M* = 1.58, *SD* = 2.43, mean difference -0.03 [95% CI -0.59 - 0.52], *P* = .90), medication use [*M* = 1.73, *SD* = 2.52 vs *M* = 2.02, *SD* = 3.49, mean difference 0.29 [95% CI -0.54 - 0.98], *P* = .42), or traditional healer engagements [*M* = 0.05, *SD* = 0.31 vs *M=* 0.16, *SD* = 0.88, mean difference 0.12 [95% CI -0.03 - 0.27], *P* = .12).

*Secondary Analyses of Women Meeting Criteria for Distress and Impairment (regardless of GBV status)*

Secondary analyses were conducted on all women who met eligibility criteria, including those who had (n = 421) and had not (n = 97) experienced GBV; this total sample comprised 518 women who were randomized to either PM+ (n = 257) or enhanced usual care (n = 261). TABLE S1 presents the participant characteristics. Planned comparisons of women in the PM+ and enhanced usual care conditions indicates that these two groups did not differ on any pretreatment factors. Comparable proportions of women were assessed at posttreatment (PM+, 205, 79.77%; EUC 214, %) and follow-up (PM+,189 [73.54%]; EUC, 195, 81.99%).

*Primary Outcome*

TABLE S2 provides the estimated mean scores for GHQ-12. Both groups displayed marked reductions in severity of psychological distress over time. The between-treatment difference at 3-month follow-up (relative to baseline) was 1.45 (95% CI, 0.07 - 2.82; *P* = .04), indicating that PM+ to greater longer-term reductions in psychological distress than EUC. Fewer women in PM+ met the criterion for psychological morbidity on the GHQ-12 at posttreatment (36.10% vs. 55.61%; OR, 2.22 [95% CI, 1.77-2.78], *P* = .001) and follow-up (27.51% vs. 36.41%; OR, 1.48 [95% CI, 1.15-1.90], *P* = .002). At follow-up there was a small between-condition effect size (0.26 [95% CI, 0.01 – 0.48]), indicating comparable gains achieved by PM+ and EUC.

*Secondary Outcomes*

PCL, WHODAS, PSYCHLOPS, and LEC scores are also displayed in TABLE S2. In terms of disability, the between-treatment difference at 3-month follow-up on the WHODAS was 0.76 (95% CI, -0.79-2.31; *P* = .34), indicating that PM+ and EUC led to comparable reductions functional impairment. At follow-up there was a small between-condition effect size (0.10 [95% CI, -0.11 – 0.31]), indicating comparable gains achieved by PM+ and EUC.

In terms of PTSD severity, the between-treatment difference at 3-month follow-up was 2.01 (95% CI, -1.56-5.57; *P* = .27), indicating that PM+ and EUC led to comparable reductions in PTSD symptoms. At follow-up there was a small between-condition effect size (0.10; 95% CI, -0.08 – 0.29), indicating comparable gains achieved by PM+ and EUC.

Regarding personalised outcomes as measured by the PSYCHLOPS, the between-treatment difference at 3-month follow-up was 1.34 (95% CI, 0.25-2.44; *P* = .02), indicating that PM+ led to a greater reduction in personally identified problems than EUC. There was a moderate between-treatment effect size in favor of PM+ (0.41; 95% CI, 0.07 – 0.75).

In terms of scores on the exposure to life stressors, the between-treatment difference at 3-month follow-up was -.16 (95% CI, -098 – 0.65; *P* = .69), indicating no difference in exposure to stressful life events during the period of the study across the two conditions. There was a very small between-treatment effect size (0.03 [95% CI, -0.23 – 0.15]).

Regarding service utilization, there were no differences between PM+ and EUC conditions at the 3-month follow-up on whether there had been a hospital admission [2.12% vs 2.56%, OR 1.20 [95% CI, 0.56 – 2.60], *P* = .64), or the number of out-patient consultations (*M* 1.59, *SD* 2.39 vs *M* 1.58, *SD* 2.39, mean difference -0.01 [95% CI -0.51 to 0.48], *P* = .95), medication use [*M* 1.90, *SD* 2.90 vs *M* 1.88, *SD* 3.29, mean difference -0.02 [95% CI -0.66 to 0.62], *P* = .95], or traditional healer engagements (*M* 0.08, *SD* 0.59 vs *M* 0.18, *SD* 0.91, mean difference 0.10 [95% CI -0.05 to 0.25], *P* = .20).

TABLE S1. Participant Characteristics and Trauma Exposure

|  | PM+  (n = 257) | Enhanced Usual Care  (n = 261) |
| --- | --- | --- |
| Age, mean (SD), y | 35.3±14.0 | 36.6±13.3 |
| Education, mean (SD) y | 8.6±3.7 | 8.3±4.24 |
| Marital status – no. (%) |  |  |
| Single | 34 (13.2) | 40 (15.3) |
| Married | 149 (58.0) | 140 (53.6) |
| Divorced/Separated | 52 (20.2) | 52 (19.9) |
| Widowed | 22 (8.6) | 29 (11.1) |
| Working – no. (%) | 126 (49.0) | 130 (49.8) |
| Trauma exposure – no. (%) |  |  |
| Disaster | 137 (53.3) | 122 (46.7) |
| Fire | 145 (56.4) | 133 (51.0) |
| Motor vehicle accident | 134 (52.1) | 131 (50.2) |
| Serious Accident | 110 (42.8) | 119 (45.6) |
| Chemical exposure | 75 (29.2) | 80 (30.6) |
| Physical assault | 173 (67.3) | 165 (63.2) |
| Assault by weapon | 113 (44.0) | 103 (39.5) |
| Sexual assault | 67 (26.1) | 79 (30.3) |
| Unwanted sexual experience | 69 (26.8) | 68 (26.0) |
| War exposure | 72 (28.0) | 69 (26.4) |
| Kidnapped | 48 (18.7) | 42 (16.1) |
| Life-threatening illness | 125 (48.6) | 119 (45.6) |

TABLE S1. Participant Characteristics and Trauma Exposure

|  | PM+  (n = 257) | Enhanced Usual Care  (n = 261) |
| --- | --- | --- |
| Witness violent death | 111 (43.2) | 107 (41.0) |
| Unexpected death of loved one | 190 (73.9) | 194 (74.3) |
| Intimate partner violence | 168 (65.4) | 164 (62.8) |
| Baseline Psychopathology Scores |  |  |
| GHQ | 19.0±5.8 | 18.7±5.8 |
| PCL | 32.7±20.1 | 30.7±19.2 |
| WHODAS | 27.9±7.7 | 27.3±7.4 |
| PSYCHLOPS | 16.6±3.2 | 16.4±3.2 |
| Violence Against Women – no. (%) |  |  |
| Slapped | 146 (56.8) | 148 (56.7) |
| Pushed | 99 (38.5) | 100 (38.3) |
| Punched | 100 (38.9) | 106 (40.6) |
| Beaten up | 104 (40.5) | 110 (42.1) |
| Choked/burnt | 46 (17.9) | 53 (20.3) |
| Used a weapon | 63 (24.5) | 69 (26.4) |
| Forced sex | 85 (33.1) | 71 (27.2) |

Table S2. Estimated Mean Scores for Primary and Secondary Outcome Measures at Baseline, Posttreatment, and 3-Month Follow-Up

| Outcomes | PM+  (n = 257) | Enhanced Usual Care  (n = 261) | *P*  Value | Effect Size or Odds Ratio  (95% CI) |
| --- | --- | --- | --- | --- |
| Primary outcome: psychological morbidity scores on GHQ-12 |  |  |  |  |
| Baseline | 19.1 (18.3 - 19.9) | 18.7 (17.8 - 19.5) |  |  |
| Posttreatment | 11.2 (10.3 - 12.2) | 13.4 (12.5 - 14.3) | .001 | 0.45 (0.20 - 0.68) |
| 3-month follow-up | 9.5 (8.6 - 10.4) | 10.5 (9.6 - 11.4) | .04 | 0.26 (0.01 - 0.48) |
| Secondary Outcomes |  |  |  |  |
| Self-reported PTSD severity on the PCL |  |  |  |  |
| Baseline | 32.8 (30.1 - 35.6) | 30.9 (28.1 - 33.7) |  |  |
| Posttreatment | 9.7 (7.4 - 12.1) | 13.2 (10.9 - 15.6) | .003 | 0.28 (0.10 - 0.47) |
| 3-month follow-up | 7.3 (5.2 - 9.3) | 7.3 (5.3 - 9.4) | .27 | 0.10 (-.08 - 0.29) |
| Outcomes | PM+  (n = 209) | Enhanced Usual Care  (n = 212) | *P*  Value | Effect Size or Odds Ratio  (95% CI) |
| Functional impairment on the WHODAS |  |  |  |  |
| Baseline | 27.9 (26.9 – 29.0) | 27.3 (26.3 - 28.4) |  |  |
| Posttreatment | 18.5 (17.3 - 19.6) | 20.3 (19.1 - 21.4) | .003 | 0.32 (0.11 - 0.54) |
| 3-month follow-up | 16.9 (15.8 - 17.9) | 17.0 (16.0 - 18.0) | .34 | 0.10 (-.11 - 0.31) |
| PSYCHLOPS |  |  |  |  |
| Baseline | 16.6 (16.0 - 17.1) | 16.4 (15.9 - 17.0) |  |  |
| Posttreatment | 9.8 (8.9 - 10.6) | 12.1 (11.3 - 12.9) | .001 | 0.76 (0.44 - 1.08) |
| Follow-up | 8.9 (8.0 - 9.7) | 10.1 (9.2 – 11.0) | .02 | 0.41 (0.08 - 0.75) |
| Life Events Checklist |  |  |  |  |
| Baseline | 8.4 (7.9 - 8.9) | 8.0 (7.5 - 8.6) |  |  |
| 3-month follow-up | 7.5 (6.9 - 8.1) | 6.8 (6.9 to 8.1) | .69 | 0.03 (-0.23 - 0.15) |

Abbreviations. GHQ-12 = General Health Questionnaire-12 (subscale score range: 0-36; higher scores indicate elevated anxiety or depression, respectively); WHODAS = WHO Disability Adjustment Scale (total score range: 0-48; higher scores indicate more severe impairment); PCL = Posttraumatic Stress Disorder Checklist (total score range: 0-80; higher scores indicate more severe PTSD severity); PSYCHLOPS = Personalized Outcome Profiles (total score range: 0-20; higher scores indicate poorer outcome).
