# Supplementary material for: Effectiveness of a brief behavioural intervention on psychological distress among women with a history of gender-based violence in urban Kenya: A randomised clinical trial
Source: PLoS Med. 2017 Aug 15;14(8):e1002371. doi: 10.1371/journal.pmed.1002371 (PMC5557357; doi:10.1371/journal.pmed.1002371)
Supplement: S4 Text — (PDF) [file pmed.1002371.s004.pdf]

S4 Text: ETHICS APPROVAL

World Health Organization

Research Ethics Review Committee (WHO ERC)

20, AVENUE APPIA – CH-1211 GENEVA 27 – SWITZERLAND – HTTP://INTRANET.WHO.INT/HOMES/RPC/ERC – HTTP://WWW.WHO.INT/RPC/RESEARCH_ETHICS

WHO ERC
Review Summary - Amendment

Protocol ID: RPC656
Protocol Title: Problem Management Plus (PM+) in the treatment of common mental disorders in women affected by adversity in urban Kenya.
Version: 2 Dated: 03.04.2014
WHO Responsible Staff Member: van Ommeren, Mark
Responsible Unit: NMH/MSD

This protocol received final approval by ERC on 30/05/2014. The unit has modified the protocol and requests the approval of the Amendments proposed. The outcome of the review is as follows:

1. It is proposed to reimburse participants to the equivalent of 4USD per assessment -for the pre and post assessments, and the equivalent of 8 USD for the 3-months follow-up. Please discuss whether the reimbursement proposed is in accordance with local wedges.
2. The ERC welcomed the fact that a local independent advisory board will review SAEs within 48 hours. Please submit the composition of the board when established and its terms of reference.
3. Please describe where the interviews will take place. Since the tool is intended to be used in communities where the access to mental health services may be limited, the protocol should clearly specify whether the RCT will be community or health institution based.
4. Please specify whether interviewers will be assessed psychologically. It would be important to ensure they do not suffer from stress and trauma/depression to participate as interviewers of the study.
5. Please confirm that training and supervision are included in the budget. It would be important to discuss in the protocol the type of supervision that will be offered to interviewers.

Based on these considerations, protocol **Version 3 Dated 12 December 2014** is Conditionally approved. Final approval is contingent on the submission of the responses to the points listed above and their incorporation (if pertinent) to the protocol.

Chairperson........................... M Gomes   Date.................................. 16th February 2015

Name: Melba Gomes/ ~~Nigel Rollins /Emilie Alirol~~

The responses provided were considered satisfactory. Protocol Version 4, Dated 12.03.2015 is approved.

M Gomes
1.4.2015

NOTE
Any changes to the proposal or to the attachments (informed consent/ questionnaires etc.) should be approved by ERC before being implemented.
